# Fructose-Asparagine Is a Primary Nutrient during Growth of *Salmonella* in the Inflamed Intestine

**DOI:** 10.1371/journal.ppat.1004209

**Published:** 2014-06-26

**Authors:** Mohamed M. Ali, David L. Newsom, Juan F. González, Anice Sabag-Daigle, Christopher Stahl, Brandi Steidley, Judith Dubena, Jessica L. Dyszel, Jenee N. Smith, Yakhya Dieye, Razvan Arsenescu, Prosper N. Boyaka, Steven Krakowka, Tony Romeo, Edward J. Behrman, Peter White, Brian M. M. Ahmer

**Affiliations:** 1 Department of Microbiology, The Ohio State University, Columbus, Ohio, United States of America; 2 Department of Medical Microbiology and Immunology, Faculty of Medicine, Mansoura University, Mansoura, Egypt; 3 Center for Microbial Pathogenesis, The Research Institute at Nationwide Children's Hospital, Columbus, Ohio, United States of America; 4 Department of Microbial Infection and Immunity, The Ohio State University, Columbus, Ohio, United States of America; 5 Department of Veterinary Biosciences, The Ohio State University, Columbus, Ohio, United States of America; 6 Department of Internal Medicine, The Ohio State University, Columbus, Ohio, United States of America; 7 Department of Microbiology and Cell Science, University of Florida, Gainesville, Florida, United States of America; 8 Department of Chemistry and Biochemistry, The Ohio State University, Columbus, Ohio, United States of America; University of California, Davis, United States of America

## Abstract

*Salmonella enterica* serovar Typhimurium (*Salmonella*) is one of the most significant food-borne pathogens affecting both humans and agriculture. We have determined that *Salmonella* encodes an uptake and utilization pathway specific for a novel nutrient, fructose-asparagine (F-Asn), which is essential for *Salmonella* fitness in the inflamed intestine (modeled using germ-free, streptomycin-treated, ex-germ-free with human microbiota, and IL10^−/−^ mice). The locus encoding F-Asn utilization, *fra*, provides an advantage only if *Salmonella* can initiate inflammation and use tetrathionate as a terminal electron acceptor for anaerobic respiration (the *fra* phenotype is lost in *Salmonella* SPI1^−^ SPI2^−^ or *ttrA* mutants, respectively). The severe fitness defect of a *Salmonella fra* mutant suggests that F-Asn is the primary nutrient utilized by *Salmonella* in the inflamed intestine and that this system provides a valuable target for novel therapies.

## Author Summary

It has long been thought that the nutrient utilization systems of *Salmonella* would not make effective drug targets because there are simply too many nutrients available to *Salmonella* in the intestine. Surprisingly, we have discovered that *Salmonella* relies heavily on a single nutrient during growth in the inflamed intestine, fructose-asparagine (F-Asn). A mutant of *Salmonella* that cannot obtain F-Asn is severely attenuated, suggesting that F-Asn is the primary nutrient utilized by *Salmonella* during inflammation. No other organism has been reported to synthesize or utilize this novel biological compound. The novelty of this nutrient and the apparent lack of utilization systems in mammals and most other bacteria suggest that the F-Asn utilization system represents a specific and potent therapeutic target for *Salmonella*.

## Introduction

*Salmonella* is a foodborne pathogen that causes significant morbidity and mortality in both developing and developed countries [1], [2]. It is widely believed that there are no undiscovered drug targets in *Salmonella enterica*, largely due to the high number of nutrients available during infection and redundancy in metabolic pathways [3], [4]. To acquire nutrients in the intestine, *Salmonella* initiates inflammation, which disrupts the microbiota and causes an oxidative burst that leads to the formation of tetrathionate [1]–[3], [5]–[7]. Tetrathionate is used as a terminal electron acceptor for the anaerobic respiration of carbon compounds that otherwise would not be metabolized [8]. One of these carbon sources is ethanolamine, which is derived from host phospholipids. Ethanolamine can be respired by *Salmonella*, but not fermented [8]. *Salmonella* actively initiates inflammation using two Type 3 Secretion Systems (T3SS), each encoded within a distinct, horizontally acquired pathogenicity island. SPI1 (*Salmonella* Pathogenicity Island 1) contributes to invasion of host cells and elicitation of inflammation in the host. SPI2 is required for survival within macrophages and contributes to intestinal inflammation. *Salmonella* strains lacking SPI1 and SPI2 cause very little intestinal inflammation [5], [6], [8], [9]. Here, we have identified fructose-asparagine (F-Asn) as another carbon source that is consumed by *Salmonella* using tetrathionate respiration during the host inflammatory response. The phenotypes of mutants lacking this utilization system are quite severe, suggesting that this is the primary nutrient utilized during *Salmonella*-mediated gastroenteritis. No other organism is known to synthesize or utilize F-Asn.

## Results

**Figure 1:**
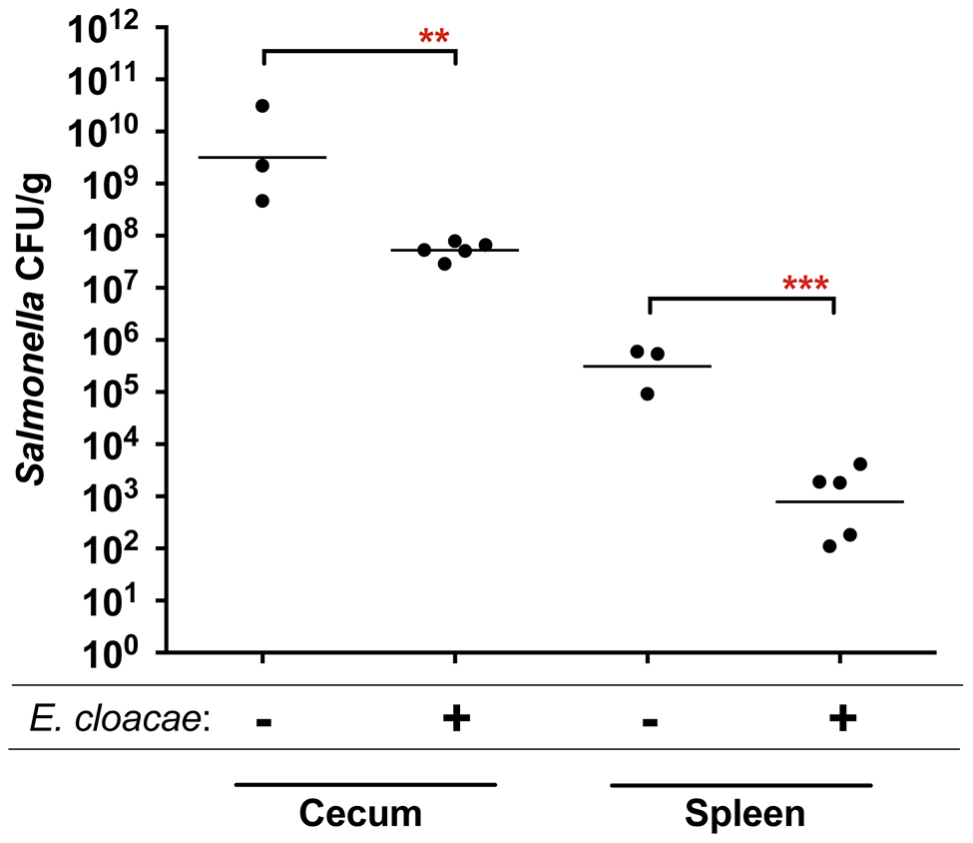
Protection of mice against *Salmonella* serovar Typhimurium strain 14028 by *Enterobacter cloacae* strain JLD400. Germ-free C57BL/6 mice were divided into two groups. One group was colonized with 10^7^ cfu of *Enterobacter cloacae* via the intragastric route (i.g.) and one group was not. One day later both groups were challenged i.g. with 10^7^ cfu of *Salmonella*. After 24 hours, the cecum and spleen were homogenized and plated to enumerate *Salmonella*. Each point represents the CFU/g recovered from one mouse with the geometric mean shown by a horizontal line. Statistical significance between select groups was determined by using an unpaired two-tailed Student *t* test. ** = P value<0.01, *** = P value<0.001.

The fructose-asparagine (F-Asn) utilization system was discovered during a genetic screen designed to identify novel microbial interactions between *Salmonella* and the normal microbiota. Transposon site hybridization (TraSH) was used to measure and compare the relative fitness of *Salmonella* transposon insertion mutants after oral inoculation and recovery from the cecum of two types of gnotobiotic mice, differing from each other by a single intestinal microbial species [10]–[15]. The two types of mice were germ-free and ex-germ-free colonized by a single member of the normal microbiota, *Enterobacter cloacae*. *E. cloacae* was chosen because it is a commensal isolate from our laboratory mice, easily cultured, genetically tractable, and it protects mice against *Salmonella* infection (Figure 1). In total, five genes conferred a greater fitness defect in the mice containing *Enterobacter* than in the germ-free mice (Table 1).

**Table 1:** Genes that are differentially required in germ-free mice and ex-germ-free mice monoassociated with *Enterobacter cloacae*.

Locus tag^a^	Symbol	Description	Germ-free mice^b^	*Enterobacter* monoassociated mice^c^	Difference^d^
STM14_2365	*sirA*	response regulator	1.88	−0.27	−2.15
STM14_3566	*barA*	hybrid sensory histidine kinase	1.09	−0.55	−1.64
STM14_4330	*fraD*	putative sugar kinase	−0.07	−1.29	−1.22
STM14_4331	*fraB*	putative phosphosugar isomerase	0.05	−1.12	−1.18
STM14_4329	*fraA*	putative transporter	−0.06	−1.23	−1.17

a The locus tag is from the *Salmonella* serovar Typhimurium strain 14028s genome (accession number NC_016856.1)

b The log2 hybridization intensity of this locus after recovery of the *Salmonella* library from germ-free mice.

c The log2 hybridization intensity of this locus after recovery of the *Salmonella* library from germ-free mice that had been previously monoassociated with *Enterobacter cloacae*.

d The difference in log2 hybridization intensity of this locus between *Enterobacter* monoassociated mice and germ-free mice.

**Figure 2:**
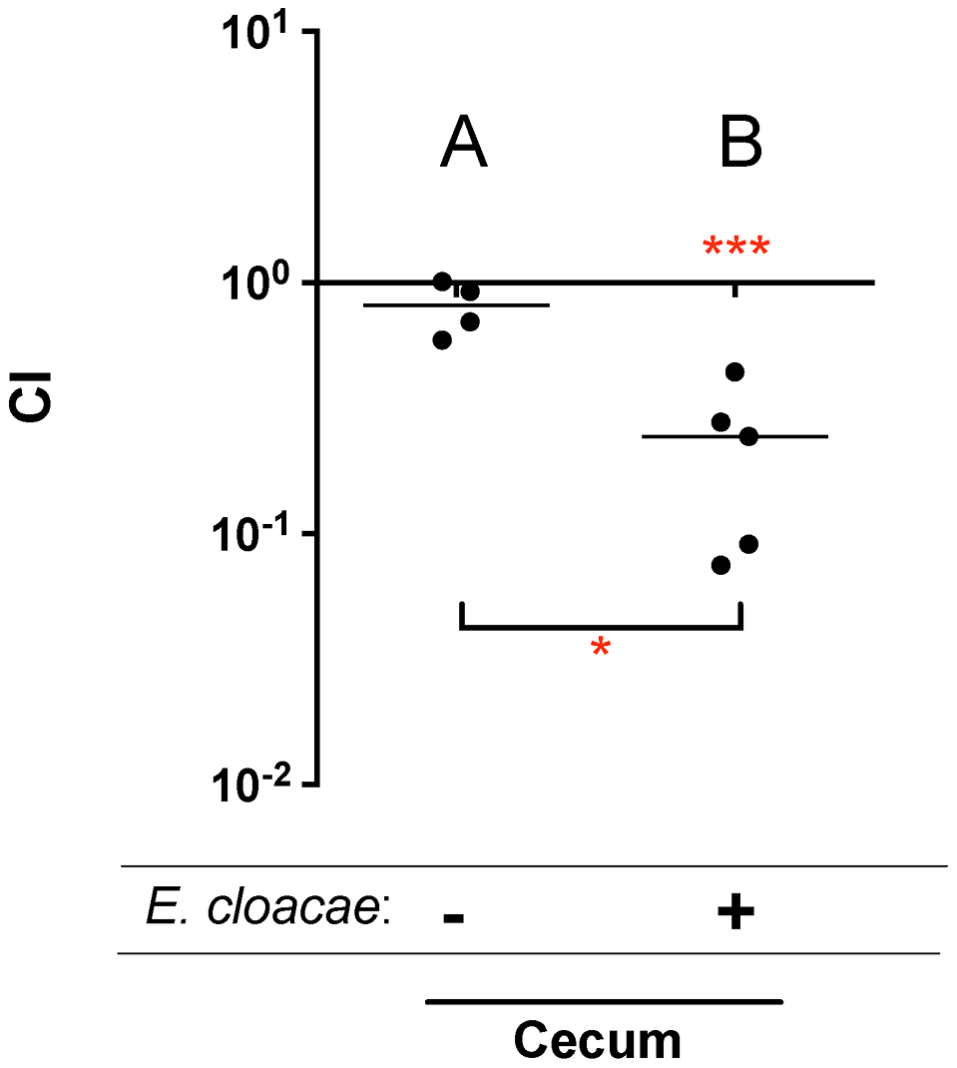
Competitive index (CI) measurements of a *sirA* mutant in mouse models. A) 10^7^ wild-type MA43 and *sirA* mutant MA45 in germ-free mice, via the intragastric route (i.g.) and recovered from the cecum after 24 hours. B) 10^7^ wild-type MA43 vs *sirA* mutant MA45 in germ-free mice mono-associated with *Enterobacter cloacae*, via the i.g. route and recovered from the cecum after 24 hours. Each point represents the CI from one mouse with the median shown by a horizontal line. Statistical significance of each group being different than 1 was determined by using a one sample Student's *t* test. Statistical significance between groups was determined using a Mann-Whitney test. * = P value<0.05, *** = P value<0.001.

Two of these genes, *barA* and *sirA* (*uvrY*), encode a two component response regulator pair that is conserved throughout the γ-proteobacteria [16]–[18]. BarA/SirA control the activity of the CsrA protein (carbon storage regulator) which coordinates metabolism and virulence by binding to and regulating the translation and/or stability of mRNAs for numerous metabolic and virulence genes including SPI1, SPI2, and *glgCAP* (glycogen biosynthesis) [17], [19], [20]. To confirm the fitness phenotype of the BarA/SirA regulatory system, we performed competition experiments in which wild-type *Salmonella* was mixed in a 1∶1 ratio with an isogenic *sirA* mutant and inoculated orally into germ-free mice and ex-germ-free mice colonized by *Enterobacter*. The results of TraSH analysis suggested that the *sirA* mutant would be at a greater growth disadvantage in *Enterobacter* mono-associated mice than in germ-free mice (Table 1). Results of the competition experiment confirmed this prediction (Figure 2).

**Figure 3:**
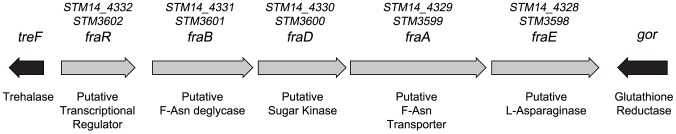
Map of the *fra* locus of *Salmonella enterica*. The five genes of the *fra* locus are shown as grey arrows. The *gor* and *treF* genes are shown as black arrows and are conserved throughout the *Enterobacteriaceae* while the *fra* locus is not, suggesting that the *fra* locus was horizontally acquired. The proposed functions and names of each gene are shown below and above the arrows, respectively. The names are based upon the distantly related *frl* locus of *E. coli*. For example, the deglycase enzyme of the *frl* locus is encoded by *frlB* so we have named the putative deglycase of the *fra* locus, *fraB*. The *fra* locus has no *frlC* homolog, while the *frl* locus does not have an asparaginase. Therefore, the name *fraC* was not used and the asparaginase was named *fraE*. The locus tags using the *Salmonella* nomenclature for strains 14028 (STM14 numbers) and LT2 (STM numbers) are shown above the gene names.

**Figure 4:**
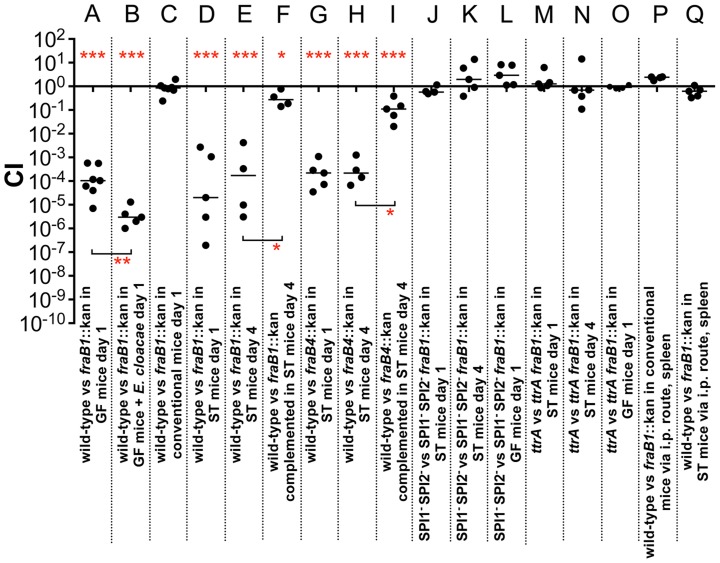
Fitness defect of a *fraB1*::kan mutant as measured by competitive index (CI) in various genetic backgrounds and mouse models. A) 10^7^ wild-type MA43 and *fraB1*::kan mutant MA59 in germ-free (GF) C57BL/6 mice, via the intragastric route (i.g.) and recovered from the cecum after 24 hours. B) 10^7^ wild-type MA43 and *fraB1*::kan mutant MA59 in germ-free C57BL/6 mice mono-associated with *Enterobacter cloacae*, via the i.g. route and recovered from the cecum after 24 hours. C) 10^9^ wild-type MA43 and *fraB1*::kan mutant MA59 in C57BL/6 conventional mice, via the i.g. route and recovered from the cecum after 24 hours. D) 10^7^ wild-type IR715 and *fraB1*::kan mutant MA59 in streptomycin-treated (ST) C57BL/6 mice, via the i.g. route and recovered from the cecum after 24 hours. E) 10^7^ wild-type IR715 and *fraB1*::kan mutant MA59 in streptomycin-treated C57BL/6 mice, via the i.g. route and recovered from the cecum after 4 days. F) Complementation of the *fraB1*::kan mutation with a plasmid encoding the entire *fra* island, pASD5006. 10^7^ ASD6090 and ASD6000 in streptomycin-treated C57BL/6 mice, via the i.g. route and recovered from the cecum after 4 days. G) 10^7^ wild-type IR715 and *fraB4*::kan mutant CS1032 in streptomycin-treated C57BL/6 mice, via the i.g. route and recovered from the cecum after 24 hours. H) 10^7^ wild-type IR715 and *fraB4*::kan mutant CS1032 in streptomycin-treated C57BL/6 mice, via the i.g. route and recovered from the cecum after 4 days. I) Complementation of the *fraB4*::kan mutation with a plasmid encoding the entire *fra* island, pASD5006.10^7^ wild-type ASD6090 and *fraB4*::kan mutant ASD6040 in streptomycin-treated C57BL/6 mice, via the i.g. route and recovered from the cecum after 4 days. J) 10^7^ *fra* ^+^ MA4301 and *fraB1*::cam mutant MA5900, both strains are a SPI1^−^ SPI2^−^ background, in streptomycin-treated C57BL/6 mice, via the i.g. route and recovered from the cecum after 24 hours. K) 10^7^ *fra* ^+^ MA4301 and *fraB1*::cam mutant MA5900, both strains are a SPI1^−^ SPI2^−^ background, in streptomycin-treated C57BL/6 mice, via the i.g. route and recovered from the cecum after 4 days. L) 10^7^ *fra* ^+^ MA4301 vs *fraB1*::cam mutant MA5900, both strains in a SPI1^−^ SPI2^−^ background, in germ-free C57BL/6 mice, via the i.g. route and recovered from the cecum after 24 hours. M) 10^7^ *fra* ^+^ MA4310 vs *fraB1*::kan mutant MA5910, both strains are a *ttrA* ^−^ background, in streptomycin-treated C57BL/6 mice, via the i.g. route and recovered from the cecum after 24 hours. N) 10^7^ *fra* ^+^ MA4310 vs *fraB1*::kan mutant MA5910, both strains are a *ttrA* ^−^ background, in streptomycin-treated C57BL/6 mice, via the i.g. route and recovered from the cecum after 4 days. O) 10^7^ *fra* ^+^ MA4310 vs *fraB1*::kan mutant MA5910, both strains are a *ttrA* ^−^ background, in germ-free C57BL/6 mice, via the i.g. route and recovered from the cecum after 24 hours. P) 10^4^ wild-type MA43 and *fraB1*::kan mutant MA59 in conventional C57BL/6 mice, via the intraperitoneal route (i.p.) and recovered from the spleen after 24 hours. Q) 10^4^ wild-type MA43 and *fraB1*::kan mutant MA59 in streptomycin-treated C57BL/6 mice, via the i.p. route and recovered from the spleen after 24 hours. Each data point represents the CI from one mouse with the median shown by a horizontal line. Statistical significance of each group being different than 1 was determined by using a one sample Student's *t* test. Statistical significance between select groups was determined using a Mann-Whitney test. * = P value<0.05, ** = P value<0.01, *** = P value<0.001.

The other three genes identified by TraSH analysis had not been characterized previously, and are located together in a putative operon. Genome annotation suggested that they encode a C4 dicarboxylate transporter, a sugar kinase, and a phosphosugar isomerase (Figure 3). A putative asparaginase lies at the end of the operon, and a separate gene upstream of the operon encodes a putative transcriptional regulator of the GntR family. These genes are not present in *E. coli* and appear to represent a horizontal acquisition inserted between the *gor* and *treF* genes at 77.7 centisomes of the *Salmonella* 14028 genome (ORFs STM14_4328 to STM14_4332). We have named these genes *fraBDAE* and *fraR* for reasons to be described below. A *fraB1*::kan mutation was constructed and tested for fitness in germ-free and *Enterobacter* colonized mice using 1∶1 competition assays against the wild-type *Salmonella*. The TraSH results suggested that this locus would exhibit a differential fitness phenotype in germ-free mice and *Enterobacter* mono-associated mice. Indeed, disruption of the *fra* locus caused a severe fitness defect in germ-free mice and a more severe defect in *Enterobacter*-colonized mice (Figure 4A, B).

### The *fra* locus confers a fitness advantage during inflammation and anaerobic respiration

Competition experiments between wild-type and the *fraB1*::kan mutant were performed as described above using conventional mice (with normal microbiota) and mice treated orally with streptomycin (strep-treated) one day earlier to disrupt the microbiota (Figure 4C, D, E). Conventional mice do not become inflamed from *Salmonella*, while strep-treated mice (or germ-free) do become inflamed [5], [6], [8], [21]–[24]. Disruption of the *fra* locus caused no fitness defect in conventional mice, but caused a severe defect in the strep-treated mice at one and four days post-infection (Figure 4C, D, E). The phenotype in strep-treated mice was confirmed by complementation (Figure 4F). It is expected that the *fraB1*::kan mutation is polar on the remainder of the *fraBDAE* operon. Therefore, the *fraB1*::kan mutation was complemented with a low copy number plasmid encoding the entire *fra* island (Figure 4F). The phenotype was confirmed again using a separately constructed mutation, *fraB4*::kan, and complementation (Figure 4G, H, I). In both instances, greater than 99% of the phenotype was restored (Figure 4F, I).

**Figure 5:**
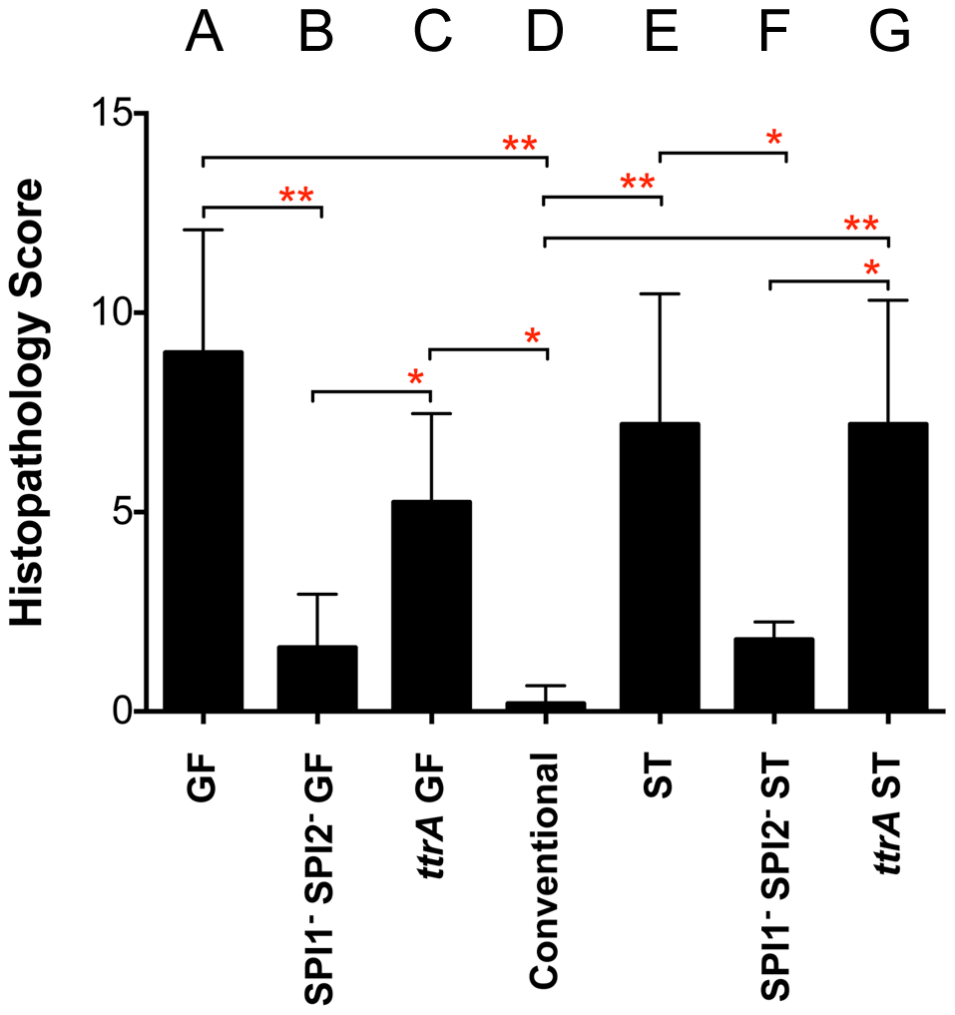
Histopathology scores of C57BL/6 mice after i.g. inoculation with *Salmonella*. All groups received 10^7^ cfu except conventional mice (D), which received 10^9^ cfu. A) Germ-free (GF) mice 24 hours post-infection with wild-type MA43 and *fraB1*::kan mutant MA59; B) GF mice 24 hours post-infection with SPI1^−^ SPI2^−^ *Salmon*ella (*fra* ^+^ MA4301 vs *fraB1*::cam mutant MA5900); C) GF mice 24 hours post-infection with *ttrA* ^−^ *Salmonella* (*fra^+^* MA4310 vs *fraB1::ka*n mutant MA5910); D) Conventional mice 24 hours post-infection with wild-type MA43 and *fraB1*::kan mutant MA59. E) Strep-treated (ST) mice 4 days post-infection with wild-type IR715 and *fraB1*::kan mutant MA59; F) ST mice 4 days post-infection with SPI1^−^ SPI2^−^ *Salmonella* (*fra* ^+^ MA4301 and *fraB1*::cam mutant MA5900; G) ST mice 4 days post-infection with *ttrA* ^−^ *Salmonella* (*fra* ^+^ MA4310 vs *fraB1*::kan mutant MA5910). Error bars represent mean+SD. Statistical significance between select groups was determined using a Mann-Whitney test. * = P value<0.05, ** = P value<0.01.

The observation of a phenotype in germ-free and strep-treated mice, but not conventional mice, suggested that *Salmonella* might require inflammation in order to acquire or utilize the *fra*-dependent nutrient source. It is known that inflammation causes the accumulation of tetrathionate in the lumen, a terminal electron acceptor that allows *Salmonella* to respire anaerobically [6]. Histopathology results confirmed that infection with *Salmonella* caused inflammation in the germ-free and strep-treated mice, but not in the conventional mice (Figure 5A, D, E). To test the hypothesis that *Salmonella* must induce inflammation for *fra* to affect the phenotype, we repeated the competition experiments in a *Salmonella* genetic background lacking SPI1 and SPI2, so that both the wild-type and the *fra* mutant would be defective for induction of inflammation. The severe fitness phenotype of the *fra* mutant was not observed in these strains (Figure 4J–L) and histopathology results confirmed that inflammation was indeed low during these experiments (Figure 5B, F).

To test the hypothesis that tetrathionate respiration was required for use of the *fra*-dependent nutrient source, the competition experiments were repeated in a *ttrA* mutant background. TtrA is part of a tetrathionate reductase, which is required for the utilization of tetrathionate as a terminal electron acceptor during anaerobic respiration [6], [25]. As in the SPI1 SPI2 background, there was no phenotype of a *fra* mutant in a *ttrA* mutant background indicating that *Salmonella* must be able to respire using tetrathionate to gain advantage from the *fra* locus (Figure 4M–O). Histopathology results confirmed the presence of moderate inflammation during these experiments (Figure 5C, G).

To determine if the *fra* locus is required during the systemic phase of disease, we performed competition experiments between the wild-type and *fra* mutant after intraperitoneal inoculation of conventional or strep-treated mice, with bacterial recovery from the spleen. The *fra* mutant had no fitness defect during systemic infection (Figure 4P, Q).

**Figure 6:**
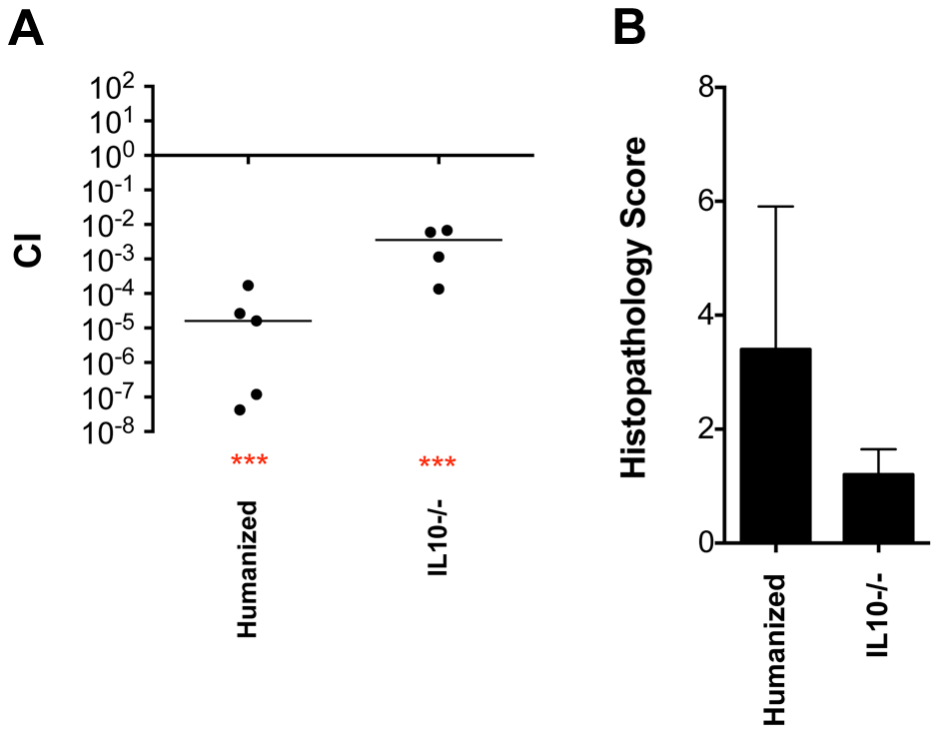
Phenotype of a *fraB1*::kan mutant in the cecum of “humanized” and IL10 knockout mice. 10^9^ wild-type IR715 vs *fraB1*::kan mutant MA59 in “humanized” Swiss Webster mice (germ free mice inoculated orally with a human fecal sample), or C57BL/6 IL10 knockout mice, as indicated, via the i.g. route and recovered from cecum on day 3 post-infection. A) Each data point represents the CI from one mouse with the median shown by a horizontal line. Statistical significance of each group being different than 1 was determined by using a one sample Student's *t* test. *** = P value<0.001. B) Histopathology scores of mice from panel A. Error bars represent mean+SD.

So far, we have seen the *fra* phenotype in C57BL/6 mice, which are mutated at the *Nramp1* locus, and this required that the mice be either germ-free or strep-treated so that *Salmonella* could induce inflammation. Ideally, we would like to determine the significance of the *fra* locus in a model that is not mutated and does not require strep-treatment or a germ-free status. It is known that humans with a complete microbiota are quickly inflamed by *Salmonella* infection while conventional mice are not, and more recently it was discovered that germ-free mice colonized with human fecal microbiota (“humanized” mice) become inflamed from *Salmonella* infection without disturbance of the gut microbiota by streptomycin [26]. Therefore, we “humanized” germ-free Swiss Webster mice, which are *Nramp1* ^+/+^, with human feces obtained from a healthy adult donor from the Ohio State University fecal transplant center. Competition experiments were then performed between wild-type and *fra* mutant *Salmonella* in these mice. Histopathology results confirmed the presence of mild inflammation during these experiments and the *fra* locus had a greater than 10,000-fold fitness phenotype (Figure 6).

IL10 knockout mice were used as another method to facilitate *Salmonella*-induced inflammation without using streptomycin [5]. Histopathology results indicated that, unexpectedly, there was not very much inflammation in these mice by day 3 post-infection although the *fra* locus still had a modest fitness phenotype (greater than 100-fold) (Figure 6). The phenotypes of the *fra* locus in IL10 knockout mice and in the humanized Swiss Webster mice demonstrate that the *fra* phenotype is not limited to germ-free or streptomycin-treated mice.

**Figure 7:**
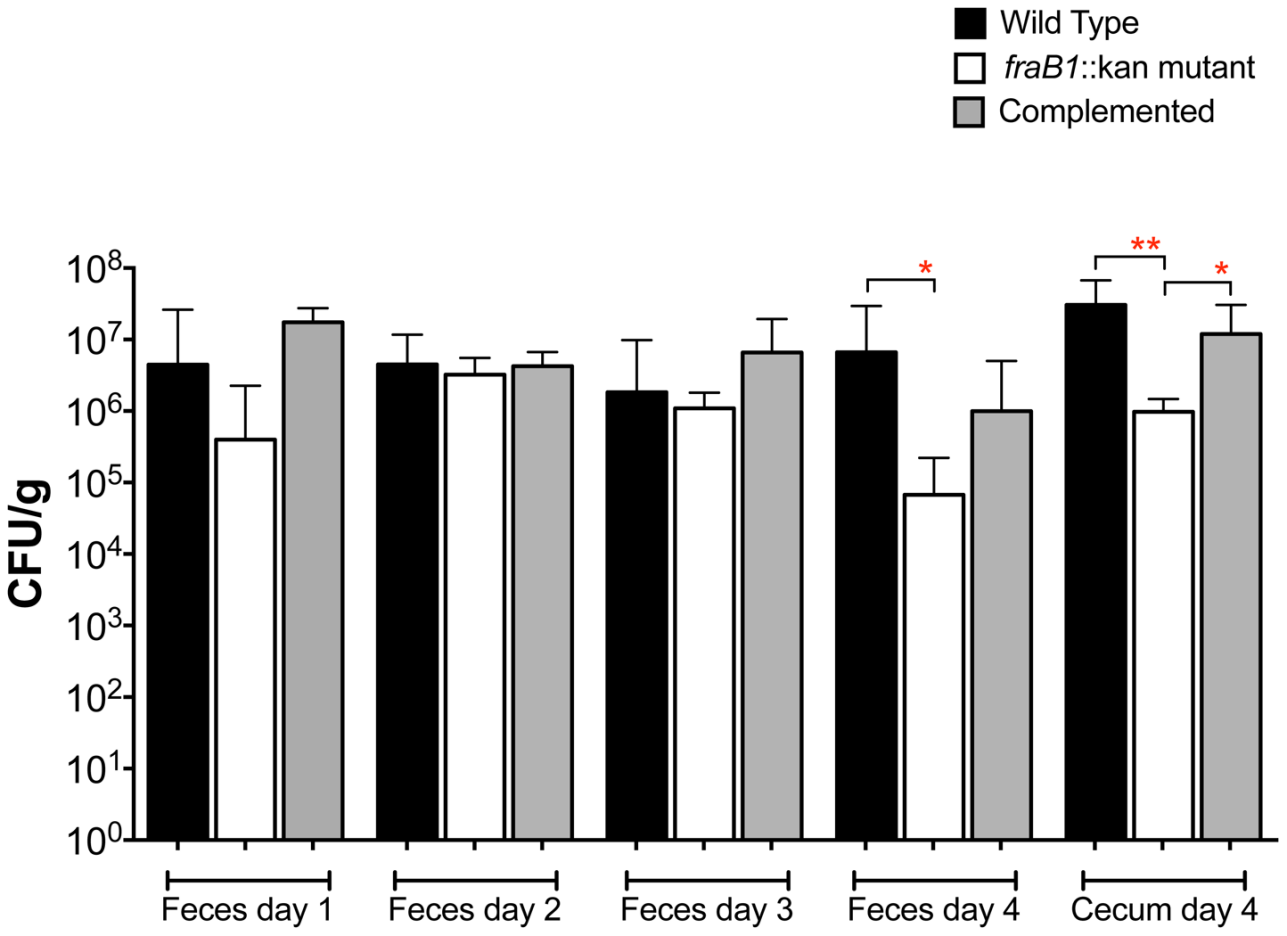
Quantitation of *Salmonella* in feces on days 1 through 4, and cecum on day 4, post-infection. Groups of five C57BL/6 mice heterozygous for *Nramp1* were orally inoculated with 10^7^ CFU of IR715 (wild-type), MA59 (*fraB1*::kan mutant), or ASD6000 (*fraB1*::kan mutant with complementation plasmid pASD5006). The geometric mean+SE is shown. Statistical significance between select groups was determined by using an unpaired two-tailed Student *t* test. * = P value<0.05, ** = P value<0.01.

Finally, to test for the possibility that these severe *fra* mutant phenotypes were the result of interaction between the wild-type and *fra* mutant during infection, we performed experiments in which strep-treated C57BL/6 *Nramp1* ^+/−^ heterozygous mice were infected separately with the wild-type, the *fra* mutant, or the complemented *fra* mutant. The strains were quantitated in the feces each day post-infection for four days at which point the mice were sacrificed and the strains were quantitated in the cecum. The *fra* mutant was recovered in 30-fold lower numbers than wild-type on the fourth day in the feces and 98-fold lower in the cecum (Figure 7). This defect was restored by complementation with the *fra* locus on a plasmid in the cecum, while in the feces the restoration did not reach statistical significance (Figure 7).

### The *fra* locus is required for growth on fructose-asparagine (F-Asn)

**Figure 8:**
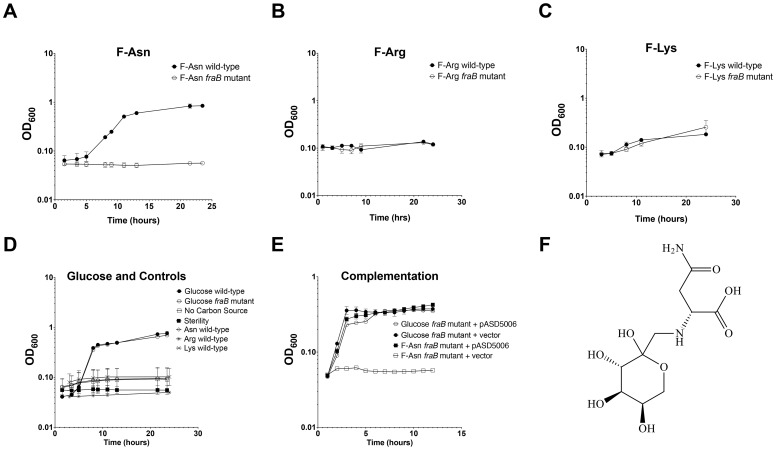
Growth of wild-type and *fraB1*::kan mutant *Salmonella* on Amadori products. Growth of wild-type MA43 and *fraB1*::kan mutant MA59 on F-Asn (A), F-Arg (B), F-Lys (C), asparagine, arginine, lysine, or glucose (D). Bacteria were grown overnight in LB at 37°C shaking, centrifuged, resuspended in water, and subcultured 1∶1000 into NCE medium containing the indicated carbon source at 5 mM. The optical density at 600 nm was then read at time points during growth at 37°C with shaking. Controls included NCE with no carbon source, and NCE with glucose that was not inoculated, as a sterility control (D). E) Complementation of a *fraB1*::kan mutation with plasmid pASD5006 encoding the *fra* island (ASD6000) or the vector control, pWSK29 (ASD6010). Each point in (A)–(E) represents the mean of three cultures with error bars indicating standard deviation. F) The structure of F-Asn (CAS#34393-27-6).

**Figure 9:**
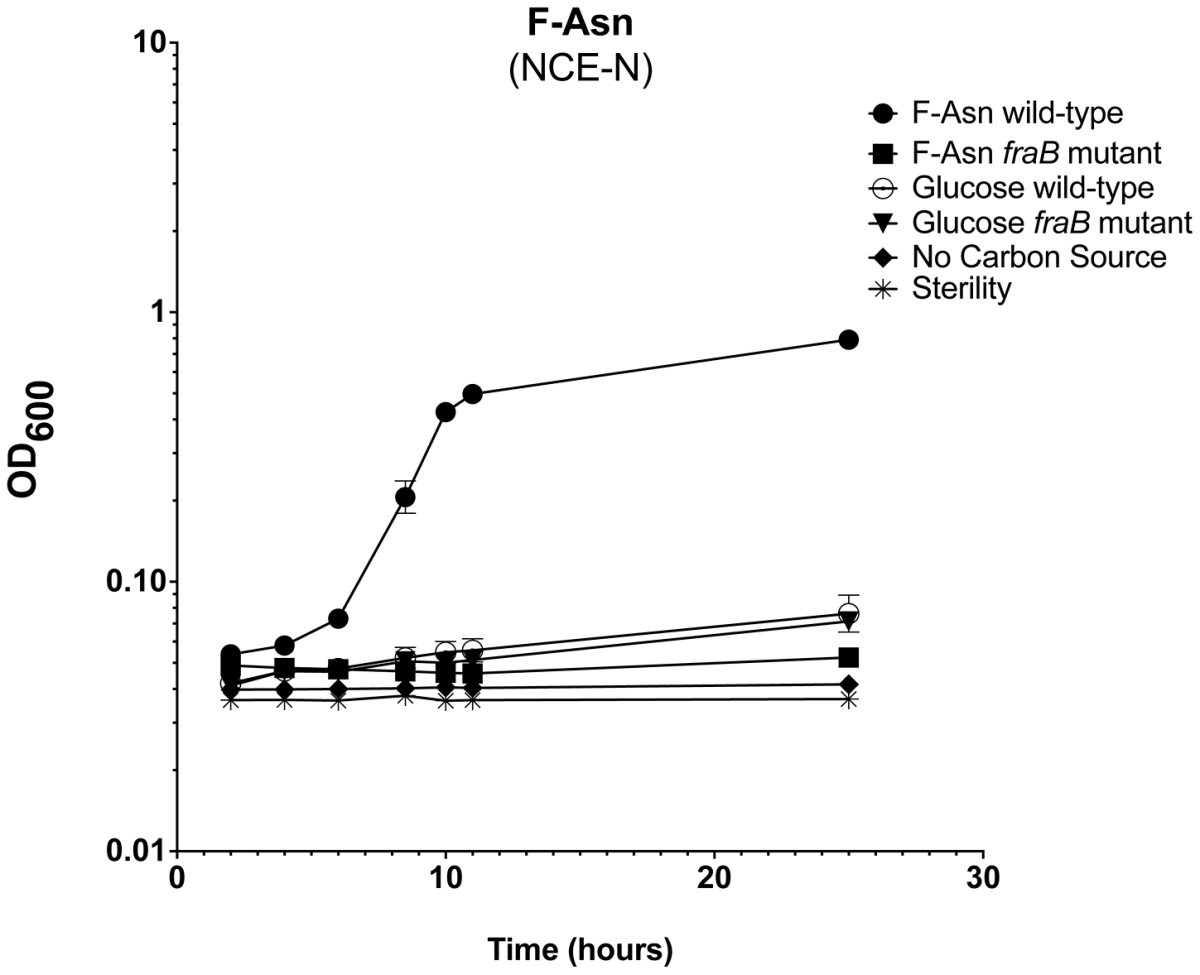
Growth of *Salmonella* on F-Asn as sole nitrogen source. Growth of wild-type MA43 and *fraB1*::kan mutant MA59 on F-Asn. Bacteria were grown overnight in LB at 37°C shaking, centrifuged, resuspended in water, and subcultured 1∶1000 into NCE medium lacking a nitrogen source (NCE-N) but containing the indicated carbon source at 5 mM. The optical density at 600 nm was then read at time points during growth at 37°C with shaking. Controls included NCE-N with no carbon source, NCE-N with 5 mM glucose, and NCE-N with glucose that was not inoculated, as a sterility control. Each point represents the mean of four cultures and error bars represent standard deviation.

FraA is homologous to the Dcu family of dicarboxylate transporters. However, authentic dicarboxylate acquisition loci do not encode a sugar kinase or phosphosugar isomerase. Furthermore, none of the dicarboxylates that we tested (malate, fumarate or succinate) provided a growth advantage to the wild-type strain vs. a *fraB1*::kan mutant, suggesting that they are not substrates of the Fra pathway. BLAST searches using the entire operon revealed that the closest homolog is the *frl* operon of *E. coli*, although the *frl* operon is at a different location within the genome and does not encode an asparaginase (and the *Salmonella fra* locus does not encode a *frlC* homolog). The products of the *E. coli frl* operon transport and degrade the Amadori product fructose-lysine (F-Lys) [27], [28]. Amadori products most often result from a spontaneous reaction between a carbonyl group (often of glucose, although numerous other compounds can also react) and an amino group of an amino acid *in vivo*, and are then referred to as non-enzymatic glycation products [29], [30]. With F-Lys and fructose-arginine (F-Arg) this can happen with the free amino acid, or the side groups of the lysine and arginine residues of a protein. In contrast, fructose-asparagine (F-Asn) can only result from reaction of glucose with the alpha amino group of free asparagine or the N-terminal asparagine of a protein. We synthesized three different Amadori products, F-Lys, F-Arg, and F-Asn and used them as sole carbon sources during growth experiments. The preparations were free of glucose but contained some free amino acid. However, control experiments demonstrated that *Salmonella* was unable to grow on any of the three amino acids alone, so these contaminants are inconsequential (Figure 8D). *Salmonella* was unable to grow on F-Arg, and grew slowly and with low yield on F-Lys (Figure 8B, C). The growth on F-Lys was independent of the *fra* locus. In contrast, *Salmonella* grew as well on F-Asn as on glucose, and growth on F-Asn was dependent upon the *fra* locus (hence the name *fra*, for fructose-asparagine utilization) (Figure 8A). A commercial source of F-Asn was obtained and it also allowed *Salmonella* to grow in a *fra*-dependent manner (structure shown in Figure 8F). Complementation of the *fraB1*::kan mutant with a plasmid encoding the *fra* island restored the ability of the mutant to grow on F-Asn (Figure 8E). In addition to serving as a sole carbon source, F-Asn, also served as sole nitrogen source (Figure 9).

**Figure 10:**
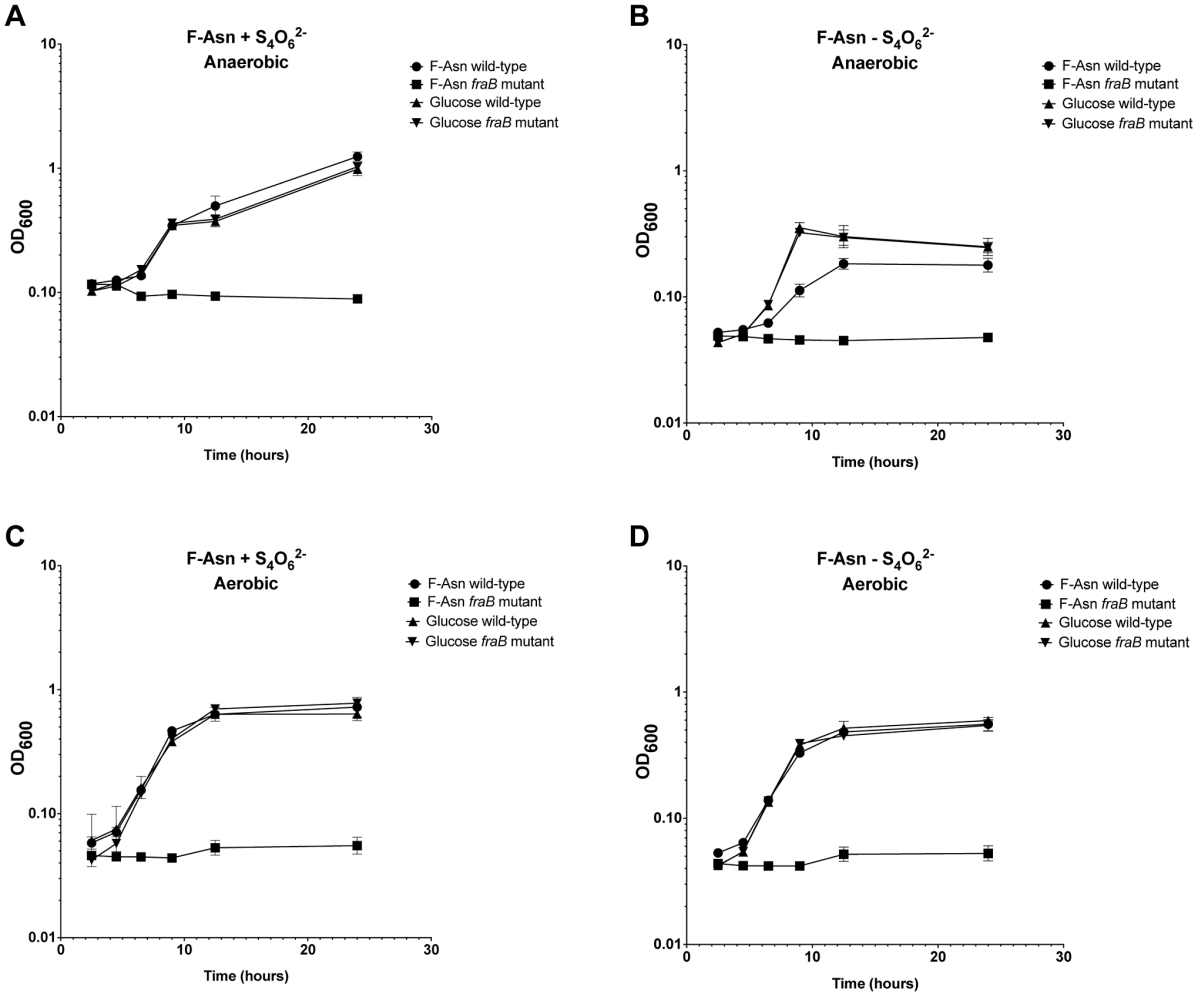
Growth of *Salmonella* on F-Asn in the presence or absence of tetrathionate or oxygen. Growth of wild-type MA43 and *fraB1*::kan mutant MA59 on 5 mM F-Asn or 5 mM glucose anaerobically (A and B) or aerobically (C and D) in the presence (A and C) or absence (B and D) of 40 mM tetrathionate (S_4_0_6_^2−^). Bacteria were grown overnight in LB at 37°C shaking, centrifuged, resuspended in water, and subcultured 1∶1000 into NCE medium containing the indicated carbon source. The optical density at 600 nm was then read at time points during growth at 37°C with shaking. Each point represents the mean of four cultures with error bars indicating standard deviation.

**Figure 11:**
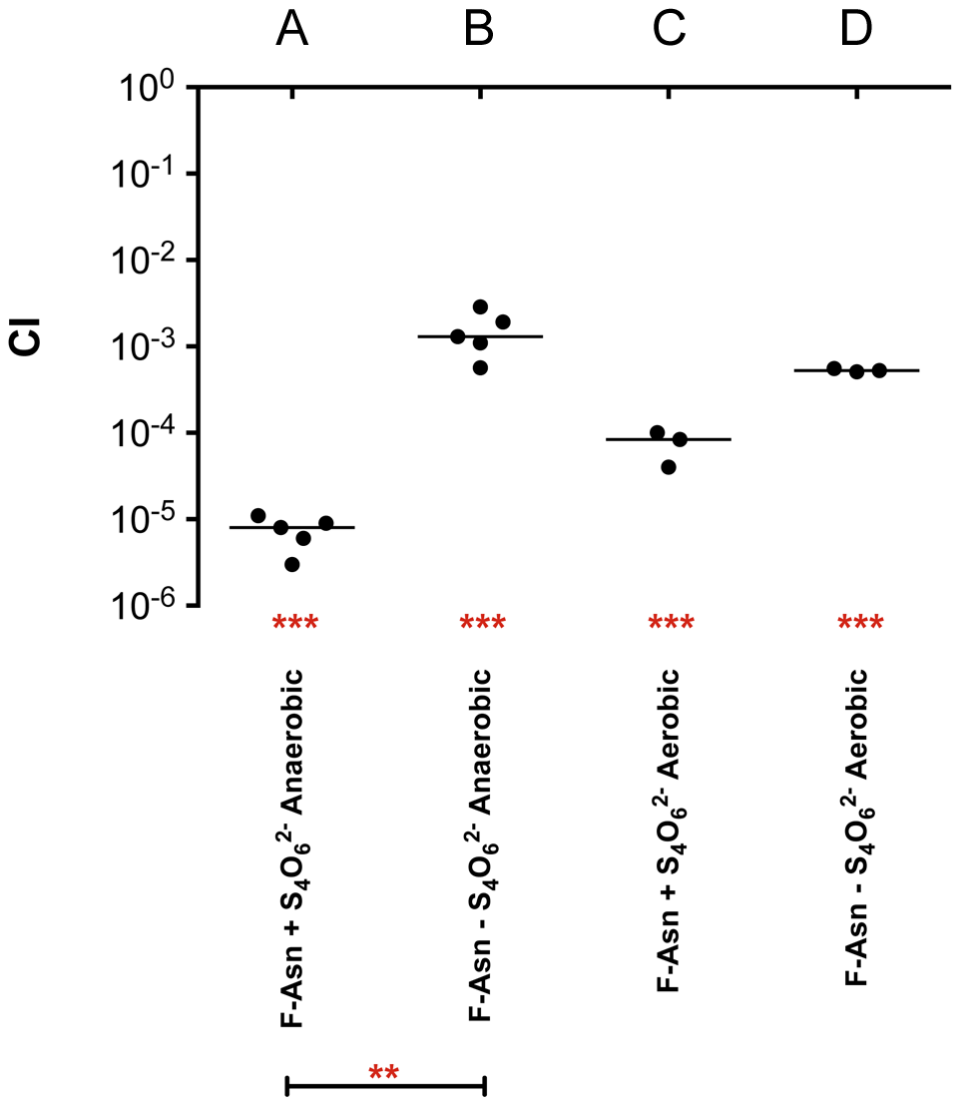
Competitive index measurements of a *fraB1*::kan mutant during *in vitro* growth. Cultures were grown overnight in LB, pelleted and washed in water, subcultured 1∶10,000 and grown for 24 hours at 37°C in NCE minimal medium containing 5 mM F-Asn, aerobically or anaerobically, in the presence or absence of tetrathionate (S_4_O_6_^2−^), as indicated. A) Anaerobic growth in the presence of tetrathionate, B) anaerobic growth in the absence of tetrathionate, C) aerobic growth in the presence of tetrathionate, D) aerobic growth in the absence of tetrathionate. Each data point represents the CI from one culture with the median shown by a horizontal line. Statistical significance of each group being different than 1 was determined by using a one sample Student's *t* test. Statistical significance between select groups was determined using a Mann-Whitney test. ** = P value<0.01, *** = P value<0.001.

Growth with F-Asn was tested under aerobic and anaerobic conditions in the presence or absence of the terminal electron acceptor tetrathionate (Figure 10). The F-Asn was utilized under all conditions, but respiratory conditions were superior with a doubling time of 1.6+/−0.1 hours aerobically with tetrathionate, 2.0+/−0.3 hours aerobically without tetrathionate, 1.9+/−0.1 hours anaerobically with tetrathionate, and 2.9+/−0.4 hours anaerobically without tetrathionate. Competition experiments in which the wild-type and *fraB1*::kan mutant were grown in the same culture were performed in minimal medium containing F-Asn. As expected, the mutant was severely attenuated during aerobic and anaerobic growth, and in the presence or absence of tetrathionate (Figure 11). The attenuation was most severe during anaerobic growth in the presence of tetrathionate.

## Discussion

The mechanisms by which microbes interact with each other in the gastrointestinal tract are largely unknown. Screening large libraries of bacterial mutants for fitness defects in animals with defined microbiota can be used to identify those genes that are only required in the presence of specific members of the microbiota [15]. In this report, we took a highly reductionist approach and screened for genes that were differentially required in germ-free mice versus ex-germ-free mice colonized with a single commensal *Enterobacter cloacae* isolate. Only five genes were differentially required, a two component response regulatory pair, *barA*/*sirA*, and three genes within the *fra* locus (Table 1). Individual *sirA* and *fraB* mutants were used to confirm the findings. The *sirA* gene was required for fitness in the presence of *E. cloacae* but not in its absence (Figure 2). The *fra* locus was required for fitness in both situations, but the phenotype was more severe in the presence of *E. cloacae* (Figure 4A, B). Thus, the differential screening strategy was successful in identifying genes that are more important in the presence of other bacteria within the gastrointestinal tract. The reason(s) that *sirA* is required in the presence, but not the absence, of *E. cloacae* is not known. It is thought that BarA detects short chain fatty acids produced by the normal microbiota and then phosphorylates SirA [31]–[34]. SirA then activates the transcription of two small RNAs, *csrB* and *csrC*, which antagonize the activity of the CsrA protein [20], [35]–[39]. The CsrA protein is an RNA-binding protein that regulates the stability and translation of hundreds of mRNAs involved with metabolism and virulence [17], [19], [40]. One possible reason that *sirA* differentially affects fitness in the two mouse models may be that the *Enterobacter*-colonized mouse offers an environment richer in carboxylic acids that act as stimuli for BarA-SirA signaling with resulting effects on metabolism and growth [31]–[34]. The fitness effects could also be due to the regulation of genes involved in the induction of inflammation and/or anareobic metabolism including SPI1, SPI2, ethanolamine utilization, and vitamin B12 biosynthesis by CsrA [19], [20], [41]–[44]. Finally, SirA or CsrA may regulate the *fra* locus itself.

**Figure 12:**
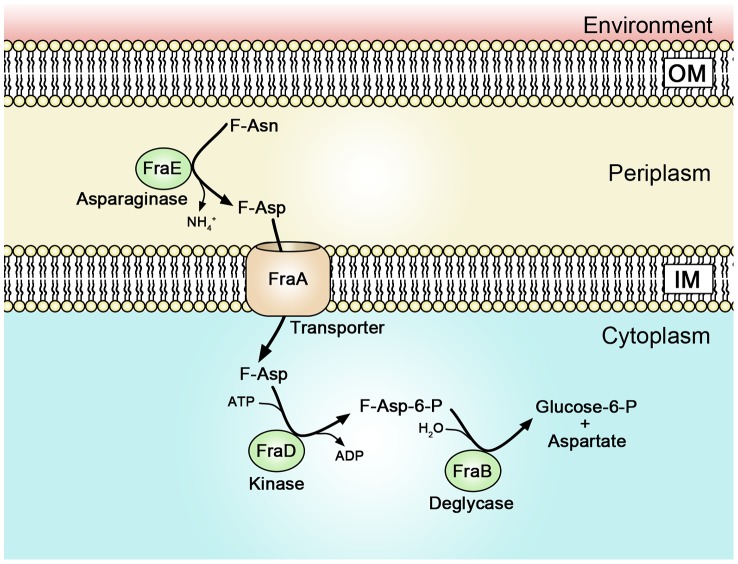
A proposed model of Fra protein localization and functions. A proteomic survey of subcellular fractions of *Salmonella* previously identified FraB (the deglycase) as cytoplasmic and FraE (the asparaginase) as periplasmic [79]. Therefore, it is possible that F-Asn is converted to F-Asp in the periplasm by the asparaginase and that the transporter and kinase actually use F-Asp as substrate rather than F-Asn. The FraD kinase of *Salmonella* shares 30% amino acid identity with the FrlD kinase of *E. coli*. FrlD phosphorylates F-Lys to form F-Lys-6-P [28]. Therefore, we hypothesize that FraD phosphorylates F-Asp to form F-Asp-6-P. The FrlB deglycase of *E. coli* shares 28% amino acid identity with FraB of *Salmonella*. The FrlB deglycase converts F-Lys-6-P to lysine and glucose-6-P [28], so we hypothesize that FraB of *Salmonella* converts F-Asp-6-P to aspartate and glucose-6-P.

The *fra* locus was annotated as a C4 dicarboxylate uptake system. However, we found that the *fra* locus played no role in the utilization of C4 dicarboxylates. BLAST searches revealed that the operon is similar to the *frl* locus of *E. coli* which is required for the utilization of fructose-lysine (F-Lys). The *frl* locus of *E. coli* has a different genomic context than the *fra* locus of *Salmonella*, and is only distantly related. We determined that the *fra* locus of *Salmonella* plays no role in the utilization of F-Lys (Figure 8C). However, the presence of an asparaginase in the *fra* locus (*fraE*), but not the *frl* locus, led us to hypothesize that F-Asn may be the correct nutrient, and indeed, this was the case. Wild-type *Salmonella* is able to grow as well on F-Asn as on glucose, and this ability is dependent upon the *fra* locus (Figures 8, 10). While the individual members of the *fra* operon have not been characterized, we hypothesize as to their functions in Figure 12. F-Asn differs from ethanolamine in that it can be fermented (Figure 10B), which would be consistent with the proposed release of glucose-6-P by FraB (Figure 12). Although F-Asn can be fermented, it only provides a fitness advantage *in vivo* when it can be respired, i.e., when tetrathionate reductase is functional (Figure 4M–O), possibly because of the much greater energy yield from respiration versus fermentation. *E. cloacae* grows very poorly on F-Asn and does not encode the *fra* locus. Therefore, *E. cloacae* likely exacerbated the *fra* phenotype of *Salmonella* by competing for other nutrients.

F-Asn is an Amadori compound (also known as a glycation product) formed by reaction between glucose in its open chain form with the alpha amino group of asparagine followed by a rearrangement that gives the fructose derivative. Until this report, no organism had been shown to synthesize or utilize F-Asn. However, in the early 2000s it was discovered that acrylamide is present in many foods, especially French fries and potato chips. F-Asn is a precursor to acrylamide. After the acrylamide discovery, numerous papers measured acrylamide concentration, and the precursor molecules, glucose and asparagine, in foods [45]–[52]. However, to the best of our knowledge, only two reports have measured the concentration of F-Asn in a few fruits and vegetables [53], [54]. The concentrations are surprisingly high, ranging between 0.1% (carrot) and 1.4% dry weight (asparagus) [54]. Factors that influence these concentrations are time, temperature, pressure, and perhaps less obviously, moisture content [55]. Any reducing sugar and any amino acid (or other amines) can form compounds analogous to F-Asn. It is important to note that these Amadori compounds are not the ultimate products since with further time and heating they decompose to a large variety of other products, some of which are responsible for a variety of flavors, and the brown color, in cooked foods [55]–[57]. In fact, glycation products form spontaneously in the human body and provide an indication of glucose concentration over time [30], [58]–[60]. A common diabetes test measures the glycation of the N-terminal valine of hemoglobin [30].

The severity of the *fra* fitness phenotype suggests that F-Asn is the primary nutrient used by *Salmonella* during growth in the inflamed intestine. For perspective, in strep-treated mice the fitness defect of a *fra* mutant is 1000-fold, while mutants unable to utilize ethanolamine or sialic acid are attenuated 10-fold and 2-fold, respectively [8], [61]. The *fra* operon was previously identified by transcription profiling as up-regulated by Fur under anaerobic conditions [62]. Other genes activated under the same conditions included ethanolamine utilization (*eut*), and *hilA*, a regulator of SPI1 expression. Both of these loci are associated with induction of inflammation or growth during inflammation [8], [62]. The *fra* locus is present among most *Salmonella* serovars, but is disrupted in serovars Typhi and Paratyphi A, consistent with the marked degradation of numerous loci involved with anaerobic respiration among these extra-intestinal serovars [63]. Interestingly, a putative *fra* locus is present in *Citrobacter rodentium* and *Citrobacter freundii*, but not in numerous other non-pathogenic *Citrobacter* species. The *frl* locus, encoding the ability to utilize F-Lys is present in *E. coli*, *Shigella*, and *Cronobacter*. It will be interesting to determine which, if any, members of the normal microbiota can compete with *E. coli* and *Salmonella* for Amadori products.

The apparent species-specificity of the F-Asn utilization system, and the severity of the fitness defect associated with mutants that cannot metabolize F-Asn, indicate that the Fra system represents a specific and valuable therapeutic target. Further studies are needed to determine the role of each gene in the *fra* locus with regard to F-Asn metabolism. Similarly, further studies are needed to determine the mechanism by which the proposed transcription factor, FraR, regulates F-Asn metabolism. These structure-function studies will facilitate small molecule drug screens targeting F-Asn utilization. It will also be interesting to determine the concentration of F-Asn and other Amadori products in a wide variety of foods, to determine if these products can affect disease susceptibility, and to explore the possibility of preventing salmonellosis or other infections by removing Amadori products from specific food products or from the diet in general. The utilization systems for many more Amadori products are likely awaiting discovery within bacterial genomes and these may play interesting roles in microbial ecology and human health.

## Materials and Methods

### Bacterial strains and media

Bacteria were grown in Luria-Bertani (LB) broth or on LB agar plates (EM Science) unless otherwise noted. The minimal medium used was NCE (no carbon E) [64] containing trace metals [25]. Chloramphenicol (cam), streptomycin (strep), or kanamycin (kan) were added at 30, 200, or 60 µg/ml, respectively, when appropriate. Fructose-asparagine was either synthesized or purchased from Toronto Research Chemicals, catalog #F792525. Anaerobic growth was performed in a Bactron 1 anaerobic chamber containing 90% N_2_, 5% CO_2_, and 5% H_2_ (Shel Lab). Strains used are described in Table 2. *Enterobacter cloacae* strain JLD400 was isolated in our laboratory by plating fecal samples from a conventional BALB/c mouse onto LB agar plates. This particular isolate was chosen because it is easy to culture and genetically manipulable (the strain can be electroporated, maintains ColE1-based plasmids, and can act as a recipient in RP4-mediated mobilization of a suicide vector used to deliver mTn*5-luxCDABE*, not shown). The species identification was performed using a Dade Microscan Walkaway 96si at the Ohio State University medical center. Additionally, genomic DNA sequences have been obtained that flank mTn*5-luxCDABE* insertions in JLD400 and these DNA sequences match the draft genome sequence of *E. cloacae* NCTC 9394.

**Table 2:** Bacterial strains and plasmids.

Strain or plasmid	Genotype or description	Source or reference
14028	wild-type *Salmonella enterica* serovar Typhimurium	American Type Culture Collection
ASD6000	MA59 *fraB1*::kan+pASD5006 (amp^r^, *fraR^+^ fraBDAE* ^+^)	This study
ASD6010	MA59 *fraB1*::kan+pWSK29 (amp^r^)	This study
ASD6040	CS1032 *fraB4*::kan+pASD5006 (amp^r^)	This study
ASD6090	IR715+pWSK29 (amp^r^)	This study
IR715	14028 nal^r^	[55], [80]
JLD400	wild-type *Enterobacter cloacae* isolated from a laboratory mouse	This study
JLD1214	14028 IG(*pagC*-STM14_1502)::cam	lambda red mutation downstream of *pagC* created using PCR primers BA1561 and BA1562, then transduced into 14028
MA43	IR715 *phoN1*::*aadA*	*phoN1*::str mutation from Helene Andrews-Polymenis collection transduced into IR715
MA45	IR715 *sirA*2::kan	IR715 transduced with P22 grown on BA736 [44], [72]
MA59	IR715 *fraB1*::kan	*fraB1*::kan mutation from Helene Andrews-Polymenis collection transduced into IR715
CS1032	IR715 *fraB4*::kan	lambda red mutation of *fraB*, created using PCR primers BA2552 and BA2553, then transduced into IR715.
MA4301	14028 Δ(*avrA-invH*)*1 ssaK*::kan	*ssaK*::kan from Micah Worley strain MJW1836 transduced into YD039 [46], [81]
MA4310	MA43 *ttrA1*::cam	*ttrA1*::cam mutation from Helene Andrews-Polymenis collection transduced into MA43
MA5900	14028 Δ(*avrA-invH*)*1 ssaK*::kan *fraB1*::cam	*fraB1*::cam mutation from Helene Andrews-Polymenis collection transduced into MA4301
MA5910	IR715 *fraB1*::kan *ttrA1*::cam	*ttrA1*::cam mutation from Helene Andrews-Polymenis collection transduced into MA59
pASD5006	pWSK29 fraRBDAE+amp^r^	This study
pWSK29	pSC101 cloning vector amp^r^	[82]
pCP20	*c*I857 λP_R_-*flp* pSC101 oriTS amp^r^ cam^r^	[66]
pKD3	FRT-*cam*-FRT oriR6K amp^r^	[66]
pKD4	FRT-*kan*-FRT oriR6K amp^r^	[66]

### *Salmonella* mutant library

A transposon mutant library was constructed in *S. enterica* serovar Typhimurium strain 14028. EZ-Tn5 <T7/kan> transposomes from Epicentre Technologies were delivered to *Salmonella* by electroporation. This transposon encodes kanamycin resistance and has a T7 RNA Polymerase promoter at the edge of the transposon pointed outward. The resulting library contains between 190,000 and 200,000 independent transposon insertions and is referred to as the JLD200k library. The insertion points of this library have been determined previously by next-generation sequencing [65]. It is estimated that approximately 4400 of the 4800 genes in the *Salmonella* genome are non-essential with regard to growth on LB agar plates [65]. Therefore, the JLD200k library is saturated with each gene having an average of 43 independent transposon insertions.

### Construction of mutations

A FRT-*kan*-FRT or FRT-*cam*-FRT cassette, generated using PCR with the primers listed in Table 3 and pKD3 or pKD4 as template, was inserted into each gene of interest (replacing all but the first ten and last ten codons) using lambda Red mutagenesis of strain 14028+pKD46 followed by growth at 37°C to remove the plasmid [66]. A temperature sensitive plasmid encoding FLP recombinase, pCP20, was then added to each strain to remove the antibiotic resistance marker [66]. The pCP20 plasmid was cured by growth at 37°C. A *fraB4*::kan mutation was constructed using primers BA2552 and BA2553 (Table 3). A FRT-*cam*-FRT was placed in an intergenic region downstream of *pagC* using primers BA1561 and BA1562 (deleting and inserting between nucleotides 1342878 and 1343056 of the 14028 genome sequence (accession number NC_016856.1) (Table 3).

**Table 3:** Oligonucleotides used.

Gene targeted	Primer name	Description	Sequence
*pagC*	BA1561	Used for lambda red mutagenesis in which the *cat* (cam^r^) gene was placed downstream of *pagC* in a neutral site using pKD3 as PCR template.	CTTCTTTACCAGTGACACGTACCTGCCTGTCTTTTCTCTTGTGTAGGCTGGAGCTGCTTCG
*pagC*	BA1562	Used for lambda red mutagenesis in which the *cat* (cam^r^) gene was placed downstream of *pagC* in a neutral site using pKD3 as PCR template.	CGAAGGCGGTCACAAAATCTTGATGACATTGTGATTAACATATGAATATCCTCCTTAG
*fra* island	BA2228	Used for amplifying the *fra* island and cloning it into a complementation vector, resulting in pASD5006.	CGCAGAATCTATCCGTCCGACAACGAAC
*fra* island	BA2229	Used for amplifying the *fra* island and cloning it into a complementation vector, resulting in pASD5006.	GCAGGTTAAGGCTCTCCGTAAAGGCCAATC
*fraB*	BA2552	Used for lambda red mutagenesis in which the *aph* (kan^r^) gene was placed within the *fraB* gene using pKD4 as PCR template.	CCTGATGTAATTAATATTCCACTTTCCACATATAGCGGCGCATATGAATATCCTCCTTAG
*fraB*	BA2553	Used for lambda red mutagenesis in which the *aph* (kan^r^) gene was placed within the *fraB* gene using pKD4 as PCR template.	AGAGGAAAGCATGATGGGTATGAAAGAGACAGTTAGCAATGTGTAGGCTGGAGCTGCTTC

### Animals

Germ-free C57BL/6 mice were obtained from Balfour Sartor of the NIH gnotobiotic resource facility at the University of North Carolina and from Kate Eaton at the University of Michigan. Germ-free Swiss Webster mice were obtained from Taconic Farms. The mice were bred and maintained under germ-free conditions in sterile isolators (Park Bioservices). Periodic Gram-staining, 16 s PCR, and pathology tests performed by the Ohio State University lab animal resources department and our own laboratory were used to confirm that the mice contained no detectable microorganisms. Conventional C57BL/6 mice were obtained from Taconic Farms. C57BL/6 mice that were heterozygous for the *Nramp1* gene were generated by breeding the standard *Nramp1* ^−/−^ mice from Taconic Farms with C57BL/6 *Nramp1* ^+/+^ mice from Greg Barton [67]. IL10 knockout mice (B6.129P2-IL10^tm1Cgn^/J) were obtained from Jackson Laboratory. Germ-free Swiss Webster mice were “humanized” by intragastric inoculation of 200 µl of human feces obtained from an anonymous healthy donor from the OSU fecal transplant center.

### Transposon Site Hybridization (TraSH)

The JLD200k transposon mutant library was grown in germ-free C57BL/6 mice in the presence or absence of *E. cloacae* strain JLD400. Four mice were inoculated intragastrically (i.g.) with 10^7^ cfu of *Enterobacter cloacae* strain JLD400 that had been grown overnight in LB shaking at 37°C. After 24 hours these mice, and an additional four germ-free mice, were inoculated with 10^7^ cfu of the JLD200k library that had been grown overnight in shaking LB kan at 37°C. Prior to inoculation of the mice, the library was spiked with an additional mutant, JLD1214, at a 1∶10∶000 ratio. This mutant contains a chloramphenicol resistance (cam^r^) gene at a neutral location in the chromosome in the intergenic region downstream of *pagC* [68]. After inoculation of mice with the spiked library, the inoculum was dilution plated to quantitate the kanamycin resistant (kan^r^) *Salmonella* library members and the cam^r^ spike strain. The remainder of the inoculum was pelleted and saved as the “input” for hybridization to microarrays. After 24 hours of infection with the JLD200k library, the mice were euthanized and organs were harvested (small intestine, cecum, large intestine, and spleen). One germ-free mouse died prior to organ harvest and was not used. All samples were homogenized and dilution plated to determine *Salmonella* counts. The remainder of the homogenate was added to 25 ml LB kan and grown overnight with shaking at 37°C to recover the library members. Each culture was then pelleted and frozen as a potential “output” sample for microarray analysis. The kan^r^ and cam^r^ colony counts recovered from each organ indicated that the spike ratio of 1∶10,000 was maintained in the intestinal samples but not in the spleen samples. This indicates that the library underwent a population bottleneck on the way to the spleen so microarray analysis of spleen samples would not be informative. The cecum samples were chosen for microarray analysis. There was one “input” sample for all arrays. There were seven separate “output” samples for the arrays; four from the cecums of *Enterobacter*-associated mice and three from germ-free mice. The output from each mouse was compared to the input on a single array. We also did a single “*in vitro*” array experiment in which the JLD200k library was grown in the presence of *Enterobacter* in liquid LB broth shaking at 37°C.

Genomic DNA was isolated from the input and output bacterial pellets. The purity and concentration of the DNA samples was assessed using a Nanodrop spectrophotometer and the quality of the DNA was assessed via agarose gel electrophoresis. All seven samples had high quality intact genomic DNA. The DNA was digested using a restriction endonuclease (*Rsa*I). Labeled RNA transcripts were obtained from the T7 promoter by *in vitro* transcription. A two-color hybridization strategy was employed. RNA transcripts from the output samples were fluorescently labeled with Cyanine-5 (Cy5, red), while the input sample was labeled with Cyanine-3 (Cy3, green). Equal molar concentrations of the output and input sample were combined and hybridized to genome-wide tiling microarrays printed commercially by Agilent Technologies. Agilent's SurePrint technology employs phosphoramadite chemistry in combination with high performance Hewlett Packard inkjet technology for in situ synthesis of 60-mer oligos. Using Agilent eArray, an easy-to-use web-based application, we were able to synthesize the arrays used by Chaudhuri *et al.* that completely tiled both the sense and anti-sense strands of the *Salmonella* SL1344 genome (AMADID 015511) [10]. Each slide contained 2 arrays, each array with 105,000 features, densely tiling the entire genome. The strain of *Salmonella* used in our experiments was 14028 and its genome sequence was only recently published (GenBank Nucleotide Accession CP001363 (complete genome) and CP001362 (plasmid)). As such, each of the 60-mer probes used by Chaudhuri *et al.* [10] were mapped to the 14028 genome using blast, and then annotated with any open reading frames (ORFs) that the probe spanned. A total of 96,749 probes mapped to the 14028 genome, with a median gap between each probe of 35 nucleotides on both strands.

After purification, the labeled samples were denatured and hybridized to the array overnight. Microarray slides were then washed and scanned with an Agilent G2505C Microarray Scanner, at 2 µm resolution. Images were analyzed with Feature Extraction 10.5 (Agilent Technologies, CA). Median foreground intensities were obtained for each spot and imported into the mathematical software package “R”, which was used for all data input, diagnostic plots, normalization and quality checking steps of the analysis process using scripts developed specifically for this analysis. In outline, the intensities were not background corrected as this has been shown to only introduce noise. The dataset was filtered to remove positive control elements and any elements that had been flagged as bad, or not present in the 14028 genome. Using the negative controls on the arrays, the background threshold was determined and all values less than this value were flagged. Finally, the Log2 ratio of output Cy5/input Cy3 (red/green) was determined for each replicate, and the data was normalized by the loess method using the LIMMA (Linear models for microarray data) package in “R” as described [69], [70]. Complete statistical analysis was then performed in “R”. Insertion mutants where the ORF is essential for survival will be selected against, and thus a negative ratio of Cy5/Cy3 (red/green) will be observed in the probes adjacent to the insertion point, resulting from higher Cy3 (green) signal from the input. Conversely, insertion mutants that were advantageous to growth in the output samples would have a positive ratio, resulting from the higher Cy5 (red) signal in the output. Mutants having no effect on growth would have equal ratios in both the output and input samples (yellow). A spreadsheet of these data is available in Dataset S1.

### Synthesis of Amadori products

We carried out the syntheses of three fructosyl amino acids with asparagine, lysine, and arginine. Hodge and Fisher's review of Amadori products was consulted as an essential starting point for synthesis [71] and the recent review by Mossine and Mawhinney of all aspects of fructose-amines was a treasure house of information [55]. We found the method of Wang et al. [72] to be the most satisfactory, however reaction times cannot be standardized and excess glucose must be removed. The reaction with asparagine is slow because asparagine is sparingly soluble in methanol. By contrast, the reaction with α-Boc-lysine is fast. Arginine is an intermediate case. Previous syntheses of F-Asn include those of Stadler et al. [46], Wang et al. [72], and Miura et al. [73]. The procedure of Stadler et al. [46] uses alkaline conditions which we thought could bring about isomerization of the sugar and racemization of the amino acid. We chose to develop the synthesis of Wang et al. [72] after trying a number of different protocols described for other amino acids [74]–[77]. Wang et al. [72], however, describe only a general method and asparagine presents some particular problems, the most important of which is the poor solubility of asparagine in methanol. We added bisulfite to the reaction mixture to reduce the formation of colored by-products [57] and finally removed excess glucose by use of a cation-exchange column according to the method of Mossine et al. [78]. Using methanol alone as solvent gives the product after refluxing for 24 hr. in approximately 10–15% yield together with recovery of about 90% of the asparagine. Although the yield is low, the starting materials are inexpensive, and the insolubility of asparagine has the advantage that F-Asn, which is quite soluble in methanol, emerges from the ion exchange column almost free of asparagine. This gave a free-flowing off-white non-hygroscopic solid. The ^1^H-NMR spectrum is complex due to the equilibrating mixture of alpha- and beta- pyranose and furanose forms [55], but integration of the upfield resonances due to asparagine and the downfield resonances due to the sugar are in the proper ratio. The material was also characterized by its specific rotation and infrared (IR) spectrum: [α]^23^_D_ −48° (c = 0.1, water) (reference [73] −40°, c = 1, water); IR (Nujol): 3350,3155, 1668, 1633, 1455, 1408, 1080 cm^−1^. Compare our preparations to results in [71], [73].

### Competition assays

Competition assays were performed in which a mutant strain was mixed in a 1∶1 ratio with an isogenic wild-type and inoculated by the intragastric (i.g.) or intraperitoneal (i.p.) route to mice. Fecal samples, intestinal sections, spleen and liver were recovered at specific times post-infection, homogenized and plated on selective plates. The wild-type and mutant strains were differentiated by antibiotic resistance. The competitive index was calculated as CI = (cfu of mutant recovered/cfu w.t. recovered)/(cfu mutant input/cfu w.t. input). If the mutant is defective compared to the wild-type it will have a CI of less than 1.

### Complementation assays

The *fra* island was PCR amplified from purified 14028 genomic DNA with primers BA2228 and BA2229 using Phusion polymerase (New England Biolabs). The PCR product was cloned into pPCR-Blunt II-TOPO (Invitrogen). The resulting clones were digested with *Eco*RI (New England Biolabs), run on an agarose gel and the 8.6 kbp *fra* fragment was gel purified (Qiagen). This purified DNA fragment was ligated into pWSK29 digested with *Eco*RI (NEB) using T4 DNA ligase (New England Biolabs) overnight at 4°C. The ligation reaction was transformed into DH5α and plated on LB containing ampicillin at 37°C. The resulting plasmid, pASD5006, or the vector control pWSK29, were electroporated into the appropriate strains.

### Ethics statement

All animal work was performed in accordance with the protocols approved by our Institutional Animal Care and Use Committee (OSU 2009A0035). The IACUC ensures compliance of this protocol with the U.S Animal Welfare Act, Guide for Care and Use of Laboratory Animals and Public Health Service Policy on Humane Care and Use of Laboratory Animals. Human fecal material was obtained from an anonymous healthy donor at the Ohio State University fecal transplant center in accordance with the protocol approved by our Institutional Review Board (OSU 2012H0367).

## Supporting Information

Dataset S1 Transposon Site Hybridization data from germ-free mice and germ-free mice monoassociated with *Enterobacter Cloacae*. As explained more fully in the Materials and Methods, a normalized Log2 ratio of output/input hybridization intensity was determined for each replicate. Insertion mutants where the ORF is essential for survival were selected against, and thus yielded a negative ratio in the probes adjacent to the insertion point. Conversely, insertion mutants that were advantageous to growth in the output samples yielded a positive ratio. The average ratio for all probes and all replicates for each locus are shown in the spreadsheet for germ-free mice and germ-free mice monoassociated with *Enterobacter cloacae*. The difference column shows the difference of the ratios for that locus between the two mouse groups to facilitate the identification of differentially required genes. The spreadsheet has two tabs, one sorted by locus tag and one sorted by difference.
(XLSX)

## References

[1] Gordon MA (2011) Invasive nontyphoidal *Salmonella* disease. Current Opinion in Infectious Diseases 24: 484–489 10.1097/QCO.0b013e32834a9980 21844803 PMC3277940

[2] Chen H-M, Wang Y, Su L-H, Chiu C-H (2013) Nontyphoid *Salmonella* Infection: Microbiology, Clinical Features, and Antimicrobial Therapy. Pediatrics & Neonatology 54: 147–152 10.1016/j.pedneo.2013.01.010 23597525

[3] Becker D, Selbach M, Rollenhagen C, Ballmaier M, Meyer TF, et al (2006) Robust *Salmonella* metabolism limits possibilities for new antimicrobials. Nature 440: 303–307 10.1038/nature04616 16541065

[4] Steeb B, Claudi B, Burton NA, Tienz P, Schmidt A, et al (2013) Parallel exploitation of diverse host nutrients enhances *Salmonella* virulence. PLoS Pathog 9: e1003301 10.1371/journal.ppat.1003301 23633950 PMC3636032

[5] Stecher B, Robbiani R, Walker AW, Westendorf AM, Barthel M, et al (2007) *Salmonella enterica* serovar Typhimurium exploits inflammation to compete with the intestinal microbiota. PLoS Biol 5: 2177–2189 10.1371/journal.pbio.0050244 17760501 PMC1951780

[6] Winter SE, Thiennimitr P, Winter MG, Butler BP, Huseby DL, et al (2010) Gut inflammation provides a respiratory electron acceptor for *Salmonella* . Nature 467: 426–429 10.1038/nature09415 20864996 PMC2946174

[7] Sekirov I, Gill N, Jogova M, Tam N, Robertson M, et al (2010) *Salmonella* SPI-1-mediated neutrophil recruitment during enteric colitis is associated with reduction and alteration in intestinal microbiota. Gut Microbes 1: 30–41 10.4161/gmic.1.1.10950 21327114 PMC3035138

[8] Thiennimitr P, Winter SE, Winter MG, Xavier MN, Tolstikov V, et al (2011) Intestinal inflammation allows *Salmonella* to use ethanolamine to compete with the microbiota. Proc Natl Acad Sci USA 108: 17480–17485 10.1073/pnas.1107857108 21969563 PMC3198331

[9] Hapfelmeier S, Stecher B, Barthel M, Kremer M, Müller AJ, et al (2005) The *Salmonella* pathogenicity island (SPI)-2 and SPI-1 type III secretion systems allow *Salmonella* serovar typhimurium to trigger colitis via MyD88-dependent and MyD88-independent mechanisms. J Immunol 174: 1675–1685. 15661931 10.4049/jimmunol.174.3.1675

[10] Chaudhuri RR, Peters SE, Pleasance SJ, Northen H, Willers C, et al (2009) Comprehensive identification of *Salmonella enterica* serovar Typhimurium genes required for infection of BALB/c mice. PLoS Pathog 5: e1000529 10.1371/journal.ppat.1000529 19649318 PMC2712085

[11] Santiviago CA, Reynolds MM, Porwollik S, Choi SH, Long F, et al (2009) Analysis of pools of targeted *Salmonella* deletion mutants identifies novel genes affecting fitness during competitive infection in mice. PLoS Pathog 5: e1000477 10.1371/journal.ppat.1000477 19578432 PMC2698986

[12] Lawley TD, Chan K, Thompson LJ, Kim CC, Govoni GR, et al (2006) Genome-wide screen for *Salmonella* genes required for long-term systemic infection of the mouse. PLoS Pathog 2: e11. 16518469 10.1371/journal.ppat.0020011 PMC1383486

[13] Badarinarayana V, Estep PW3, Shendure J, Edwards J, Tavazoie S, et al (2001) Selection analyses of insertional mutants using subgenic-resolution arrays. Nat Biotechnol 19: 1060–1065. 11689852 10.1038/nbt1101-1060

[14] Sassetti CM, Boyd DH, Rubin EJ (2001) Comprehensive identification of conditionally essential genes in mycobacteria. Proc Natl Acad Sci USA 98: 12712–12717 10.1073/pnas.231275498 11606763 PMC60119

[15] Goodman AL, McNulty NP, Zhao Y, Leip D, Mitra RD, et al (2009) Identifying genetic determinants needed to establish a human gut symbiont in its habitat. Cell Host Microbe 6: 279–289 10.1016/j.chom.2009.08.003 19748469 PMC2895552

[16] Teplitski M, Ahmer BMM, Prüss BM (2005) The control of secondary metabolism, motility, and virulence by the two-component regulatory system BarA/SirA of *Salmonella* and other γ-proteobacteria. Research Signpost 26.

[17] Romeo T, Vakulskas CA, Babitzke P (2013) Post-transcriptional regulation on a global scale: form and function of Csr/Rsm systems. Environ Microbiol 15: 313–324 10.1111/j.1462-2920.2012.02794.x 22672726 PMC3443267

[18] Lapouge K, Schubert M, Allain FH-T, Haas D (2008) Gac/Rsm signal transduction pathway of gamma-proteobacteria: from RNA recognition to regulation of social behaviour. Mol Microbiol 67: 241–253 10.1111/j.1365-2958.2007.06042.x 18047567

[19] Lawhon SD, Frye JG, Suyemoto M, Porwollik S, McClelland M, et al (2003) Global regulation by CsrA in *Salmonella typhimurium* . Mol Microbiol 48: 1633–1645. 12791144 10.1046/j.1365-2958.2003.03535.x

[20] Martínez LC, Yakhnin H, Camacho MI, Georgellis D, Babitzke P, et al (2011) Integration of a complex regulatory cascade involving the SirA/BarA and Csr global regulatory systems that controls expression of the *Salmonella* SPI-1 and SPI-2 virulence regulons through HilD. Mol Microbiol 80: 1637–1656 10.1111/j.1365-2958.2011.07674.x 21518393 PMC3116662

[21] Barthel M, Hapfelmeier S, Quintanilla-Martinez L, Kremer M, Rohde M, et al (2003) Pretreatment of mice with streptomycin provides a *Salmonella enterica* serovar Typhimurium colitis model that allows analysis of both pathogen and host. Infect Immun 71: 2839–2858. 12704158 10.1128/IAI.71.5.2839-2858.2003 PMC153285

[22] Woo H, Okamoto S, Guiney D, Gunn JS, Fierer J (2008) A model of *Salmonella* colitis with features of diarrhea in SLC11A1 wild-type mice. PLoS ONE 3: e1603 10.1371/journal.pone.0001603 18270590 PMC2225501

[23] Garner CD, Antonopoulos DA, Wagner B, Duhamel GE, Keresztes I, et al (2009) Perturbation of the small intestine microbial ecology by streptomycin alters pathology in a *Salmonella enterica* serovar Typhimurium murine model of infection. Infect Immun 77: 2691–2702 10.1128/IAI.01570-08 19433544 PMC2708583

[24] Kaiser P, Diard M, Stecher B, Hardt W-D (2012) The streptomycin mouse model for *Salmonella* diarrhea: functional analysis of the microbiota, the pathogen“s virulence factors, and the host”s mucosal immune response. Immunol Rev 245: 56–83 10.1111/j.1600-065X.2011.01070.x 22168414

[25] Price-Carter M, Tingey J, Bobik TA, Roth JR (2001) The Alternative Electron Acceptor Tetrathionate Supports B12-Dependent Anaerobic Growth of *Salmonella enterica* Serovar Typhimurium on Ethanolamine or 1,2-Propanediol. J Bacteriol 183: 2463–2475 10.1128/JB.183.8.2463-2475.2001 11274105 PMC95162

[26] Chung H, Pamp SJ, Hill JA, Surana NK, Edelman SM, et al (2012) Gut immune maturation depends on colonization with a host-specific microbiota. Cell 149: 1578–1593 10.1016/j.cell.2012.04.037 22726443 PMC3442780

[27] Wiame E, Van Schaftingen E (2004) Fructoselysine 3-epimerase, an enzyme involved in the metabolism of the unusual Amadori compound psicoselysine in *Escherichia coli* . Biochem J 378: 1047–1052 10.1042/BJ20031527 14641112 PMC1224009

[28] Wiame E, Delpierre G, Collard F, Van Schaftingen E (2002) Identification of a pathway for the utilization of the Amadori product fructoselysine in *Escherichia coli* . J Biol Chem 277: 42523–42529 10.1074/jbc.M200863200 12147680

[29] Zhang Q, Ames JM, Smith RD, Baynes JW, Metz TO (2009) A Perspective on the Maillard Reaction and the Analysis of Protein Glycation by Mass Spectrometry: Probing the Pathogenesis of Chronic Disease. J Proteome Res 8: 754–769 10.1021/pr800858h 19093874 PMC2642649

[30] Tessier FJ (2010) The Maillard reaction in the human body. The main discoveries and factors that affect glycation. Pathol Biol 58: 214–219 10.1016/j.patbio.2009.09.014 19896783

[31] Hung C-C, Garner CD, Slauch JM, Dwyer ZW, Lawhon SD, et al (2013) The intestinal fatty acid propionate inhibits *Salmonella* invasion through the post-translational control of HilD. Mol Microbiol 87: 1045–1060 10.1111/mmi.12149 23289537 PMC3581741

[32] Chavez RG, Alvarez AF, Romeo T, Georgellis D (2010) The physiological stimulus for the BarA sensor kinase. J Bacteriol 192: 2009–2012 10.1128/JB.01685-09 20118252 PMC2838055

[33] Huang Y, Suyemoto M, Garner CD, Cicconi KM, Altier C (2008) Formate acts as a diffusible signal to induce *Salmonella* invasion. J Bacteriol 190: 4233–4241 10.1128/JB.00205-08 18424519 PMC2446767

[34] Lawhon SD, Maurer R, Suyemoto M, Altier C (2002) Intestinal short-chain fatty acids alter *Salmonella typhimurium* invasion gene expression and virulence through BarA/SirA. Mol Microbiol 46: 1451–1464. 12453229 10.1046/j.1365-2958.2002.03268.x

[35] Romeo T (1998) Global regulation by the small RNA-binding protein CsrA and the non-coding RNA molecule CsrB. Mol Microbiol 29: 1321–1330. 9781871 10.1046/j.1365-2958.1998.01021.x

[36] Liu MY, Gui G, Wei B, Preston JF, Oakford L, et al (1997) The RNA molecule CsrB binds to the global regulatory protein CsrA and antagonizes its activity in *Escherichia coli* . J Biol Chem 272: 17502–17510. 9211896 10.1074/jbc.272.28.17502

[37] Teplitski M, Goodier RI, Ahmer BMM (2006) Catabolite repression of the SirA regulatory cascade in *Salmonella enterica* . Int J Med Microbiol 296: 449–466 10.1016/j.ijmm.2006.06.001 16949866

[38] Fortune DR, Suyemoto M, Altier C (2006) Identification of CsrC and characterization of its role in epithelial cell invasion in *Salmonella enterica* serovar Typhimurium. Infect Immun 74: 331–339 10.1128/IAI.74.1.331-339.2006 16368988 PMC1346597

[39] Martínez LC, Martínez-Flores I, Salgado H, Fernández-Mora M, Medina-Rivera A, et al (2014) In Silico Identification and Experimental Characterization of Regulatory Elements Controlling the Expression of the *Salmonella csrB* and *csrC* Genes. J Bacteriol 196: 325–336 10.1128/JB.00806-13 24187088 PMC3911252

[40] Edwards AN, Patterson-Fortin LM, Vakulskas CA, Mercante JW, Potrykus K, et al (2011) Circuitry linking the Csr and stringent response global regulatory systems. Mol Microbiol 80: 1561–1580 10.1111/j.1365-2958.2011.07663.x 21488981 PMC3115499

[41] Bustamante VH, Martínez LC, Santana FJ, Knodler LA, Steele-Mortimer O, et al (2008) HilD-mediated transcriptional cross-talk between SPI-1 and SPI-2. Proc Natl Acad Sci USA 105: 14591–14596 10.1073/pnas.0801205105 18799744 PMC2567235

[42] Altier C, Suyemoto M, Ruiz AI, Burnham KD, Maurer R (2000) Characterization of two novel regulatory genes affecting *Salmonella* invasion gene expression. Mol Microbiol 35: 635–646. 10672185 10.1046/j.1365-2958.2000.01734.x

[43] Johnston C, Pegues DA, Hueck CJ, Lee CA, Miller SI (1996) Transcriptional activation of *Salmonella typhimurium* invasion genes by a member of the phosphorylated response-regulator superfamily. Mol Microbiol 22: 715–727 10.1046/j.1365-2958.1996.d01-1719.x 8951818

[44] Ahmer BM, van Reeuwijk J, Watson PR, Wallis TS, Heffron F (1999) *Salmonella* SirA is a global regulator of genes mediating enteropathogenesis. Mol Microbiol 31: 971–982. 10048039 10.1046/j.1365-2958.1999.01244.x

[45] Vivanti V, Finotti E, Friedman M (2006) Level of acrylamide precursors asparagine, fructose, glucose, and sucrose in potatoes sold at retail in Italy and in the United States. Journal of food science 71: C81–C85.

[46] Stadler RH, Robert F, Riediker S, Varga N, Davidek T, et al (2004) In-depth mechanistic study on the formation of acrylamide and other vinylogous compounds by the Maillard reaction. J Agric Food Chem 52: 5550–5558 10.1021/jf0495486 15315399

[47] Surdyk N, Rosén J, Andersson R, Aman P (2004) Effects of asparagine, fructose, and baking conditions on acrylamide content in yeast-leavened wheat bread. J Agric Food Chem 52: 2047–2051 10.1021/jf034999w 15053550

[48] Yaylayan VA, Wnorowski A, Perez Locas C (2003) Why Asparagine Needs Carbohydrates To Generate Acrylamide. J Agric Food Chem 51: 1753–1757 10.1021/jf0261506 12617619

[49] Mottram DS, Wedzicha BL, Dodson AT (2002) Food chemistry: Acrylamide is formed in the Maillard reaction. Nature 419: 448–449 10.1038/419448a 12368844

[50] Tareke E, Rydberg P, Karlsson P, Eriksson S, Törnqvist M (2002) Analysis of Acrylamide, a Carcinogen Formed in Heated Foodstuffs. J Agric Food Chem 50: 4998–5006 10.1021/jf020302f 12166997

[51] Tareke E, Rydberg P, Karlsson P, Eriksson S, Törnqvist M (2000) Acrylamide: A Cooking Carcinogen? Chem Res Toxicol 13: 517–522 10.1021/tx9901938 10858325

[52] Elmore JS, Mottram DS (2002) Compilation of free amino acid data for various food raw materials, showing the relative contributions of asparagine, glutamine, aspartic acid and glutamic acid to the free amino acid composition. JIFSAN Acrylamide in Food Workshop, Chicago.

[53] Anet EFLJ, Reynolds TM (1957) Chemistry of non-enzymic browning. II. Reactions between Amino Acids, Organic Acids, and sugars in freeze-dried Apricots and Peaches. Aust J Chem 10: 182–191 10.1071/CH9570182

[54] Eichner K, Reutter M, Wittmann R (1994) Detection of Amadori compounds in heated foods. Thermally Generated Flavors (ACS Symposium Series 543). Parliament TH, Morello MJ, McGorrin RJ, editors, Washington D.C.: American Chemical Society, Chapter 5.

[55] Mossine VV, Mawhinney TP (2010) 1-Amino-1-deoxy-D-fructose (“fructosamine”) and its derivatives. Adv Carbohydr Chem Biochem 64: 291–402 10.1016/S0065-2318(10)64006-1 20837201

[56] Mottram DS (2007) The Maillard Reaction: Source of Flavour in Thermally Processed Foods. Flavours and Fragrances. Berlin, Heidelberg: Springer Berlin Heidelberg. pp. 269–283. doi:10.1007/978-3-540-49339-6_12.

[57] Anet EFLJ (1957) Chemistry of non-enzymic browning. II. Some Crystalline Amino Acid-Deoxy-sugars. Aust J Chem 10: 193–197 10.1071/CH9570193

[58] Bodiga VL, Eda SR, Bodiga S (2013) Advanced glycation end products: role in pathology of diabetic cardiomyopathy. Heart Fail Rev 19: 49–63 10.1007/s10741-013-9374-y 23404649

[59] Kato S, Itoh K, Ochiai M, Iwai A, Park Y, et al (2008) Increased pentosidine, an advanced glycation end-product, in urine and tissue reflects disease activity in inflammatory bowel diseases. Journal of Gastroenterology and Hepatology 23: S140–S145 10.1111/j.1440-1746.2008.05552.x 19120887

[60] Brownlee M (1995) Advanced protein glycosylation in diabetes and aging. Annu Rev Med 46: 223–234 10.1146/annurev.med.46.1.223 7598459

[61] Ng KM, Ferreyra JA, Higginbottom SK, Lynch JB, Kashyap PC, et al (2013) Microbiota-liberated host sugars facilitate post-antibiotic expansion of enteric pathogens. Nature 502: 96–99 10.1038/nature12503 23995682 PMC3825626

[62] Troxell B, Fink RC, Porwollik S, McClelland M, Hassan HM (2011) The Fur regulon in anaerobically grown *Salmonella enterica* sv. Typhimurium: identification of new Fur targets. BMC Microbiol 11: 236 10.1186/1471-2180-11-236 22017966 PMC3212961

[63] Nuccio SP, Bäumler AJ (2014) Comparative Analysis of *Salmonella* Genomes Identifies a Metabolic Network for Escalating Growth in the Inflamed Gut. MBio 5: e00929–14–e00929–14 10.1128/mBio.00929-14 24643865 PMC3967523

[64] Davis RW, Botstein D, Roth JR (1980) Advanced bacterial genetics. Cold Spring Harbor, N.Y.: Cold Spring Harbor Laboratory. 1 p.

[65] Canals R, Xia X-Q, Fronick C, Clifton SW, Ahmer BM, et al (2012) High-throughput comparison of gene fitness among related bacteria. BMC Genomics 13: 212 10.1186/1471-2164-13-212 22646920 PMC3487940

[66] Datsenko KA, Wanner BL (2000) One-step inactivation of chromosomal genes in *Escherichia coli* K-12 using PCR products. Proc Natl Acad Sci USA 97: 6640–6645 10.1073/pnas.120163297 10829079 PMC18686

[67] Arpaia N, Godec J, Lau L, Sivick KE, McLaughlin LM, et al (2011) TLR signaling is required for *Salmonella typhimurium* virulence. Cell 144: 675–688 10.1016/j.cell.2011.01.031 21376231 PMC3063366

[68] Gunn JS, Ryan SS, Van Velkinburgh JC, Ernst RK, Miller SI (2000) Genetic and functional analysis of a PmrA-PmrB-regulated locus necessary for lipopolysaccharide modification, antimicrobial peptide resistance, and oral virulence of *Salmonella enterica* serovar Typhimurium. Infect Immun 68: 6139–6146. 11035717 10.1128/iai.68.11.6139-6146.2000 PMC97691

[69] Smyth GK, Speed T (2003) Normalization of cDNA microarray data. Methods 31: 265–273. 14597310 10.1016/s1046-2023(03)00155-5

[70] Smyth GK, Yang YH, Speed T (2003) Statistical issues in cDNA microarray data analysis. Methods Mol Biol 224: 111–136 10.1385/1-59259-364-X:111 12710670

[71] Hodge JE, Fisher BE (1963) Amadori rearrangement products. Methods in Carbohydrate Chemistry 2: 99–107.

[72] Wang J, Lu Y-M, Liu B-Z, He H-Y (2008) Electrospray positive ionization tandem mass spectrometry of Amadori compounds. J Mass Spectrom 43: 262–264 10.1002/jms.1290 17935069

[73] Miura Y, Tahara S, Mizutani J (1973) Isolation and identification of 1-deoxy-1-(L-asparagino)-D-fructose formed in the autoclaved reaction medium. Agric Biol Chem 37: 2669–2670.

[74] Keil P, Mortensen HB, Christophersen C (1985) Fructosylvaline. A simple model of the N-terminal residue of human haemoglobin A1c. Acta Chem Scand, B, Org Chem Biochem 39: 191–193. 10.3891/acta.chem.scand.39b-0191 3993309

[75] Krause R, Knoll K, Henle T (2003) Studies on the formation of furosine and pyridosine during acid hydrolysis of different Amadori products of lysine. Eur Food Res Technol 216: 277–283 10.1007/s00217-002-0649-0

[76] Srinivas SM, Harohally NV (2012) Improved synthesis of lysine- and arginine-derived Amadori and Heyns products and in vitro measurement of their angiotensin I-converting enzyme inhibitory activity. J Agric Food Chem 60: 1522–1527 10.1021/jf204185y 22242891

[77] Weitzel G, Geyer H-U, Fretzdorff A-M (1957) Darstellung und Stabilität der Salze von Aminosäure-N-Glykosiden. Chem Ber 90: 1153–1161 10.1002/cber.19570900641

[78] Mossine VV, Glinsky GV, Feather MS (1994) The preparation and characterization of some Amadori compounds (1-amino-1-deoxy-D-fructose derivatives) derived from a series of aliphatic omega-amino acids. Carbohydr Res 262: 257–270. 7982218 10.1016/0008-6215(94)84183-7

[79] Brown RN, Sanford JA, Park JH, Deatherage BL, Champion BL, et al (2012) A Comprehensive Subcellular Proteomic Survey of *Salmonella* Grown under Phagosome-Mimicking versus Standard Laboratory Conditions. Int J Proteomics 2012: 123076 10.1155/2012/123076 22900174 PMC3410353

[80] Stojiljkovic I, Bäumler AJ, Heffron F (1995) Ethanolamine utilization in *Salmonella typhimurium*: nucleotide sequence, protein expression, and mutational analysis of the *cchA cchB eutE eutJ eutG eutH* gene cluster. J Bacteriol 177: 1357–1366. 7868611 10.1128/jb.177.5.1357-1366.1995 PMC176743

[81] Teplitski M, Al-Agely A, Ahmer BMM (2006) Contribution of the SirA regulon to biofilm formation in *Salmonella enterica* serovar Typhimurium. Microbiology (Reading, Engl) 152: 3411–3424 10.1099/mic.0.29118-0 17074910

[82] Wang RF, Kushner SR (1991) Construction of versatile low-copy-number vectors for cloning, sequencing and gene expression in *Escherichia coli* . Gene 100: 195–199. 2055470

